# Transposon Insertion in the *purL* Gene Induces Biofilm Depletion in *Escherichia coli* ATCC 25922

**DOI:** 10.3390/pathogens9090774

**Published:** 2020-09-22

**Authors:** Virginio Cepas, Victoria Ballén, Yaiza Gabasa, Miriam Ramírez, Yuly López, Sara Mª Soto

**Affiliations:** ISGlobal, Hospital Clínic—Universitat de Barcelona, 08036 Barcelona, Spain; virginio.cepas@isglobal.org (V.C.); victoria.ballen@isglobal.org (V.B.); yaiza.gabasa@isglobal.org (Y.G.); miriam.ramirez@isglobal.org (M.R.)

**Keywords:** biofilm, *E. coli*, *purL*, transposon insertion, curli fibers

## Abstract

Current *Escherichia coli* antibiofilm treatments comprise a combination of antibiotics commonly used against planktonic cells, leading to treatment failure. A better understanding of the genes involved in biofilm formation could facilitate the development of efficient and specific new antibiofilm treatments. A total of 2578 *E. coli* mutants were generated by transposon insertion, of which 536 were analysed in this study. After sequencing, Tn263 mutant, classified as low biofilm-former (LF) compared to the wild-type (wt) strain (ATCC 25922), showed an interruption in the *purL* gene, involved in the de novo purine biosynthesis pathway. To elucidate the role of *purL* in biofilm formation, a knockout was generated showing reduced production of curli fibres, leading to an impaired biofilm formation. These conditions were restored by complementation of the strain or addition of exogenous inosine. Proteomic and transcriptional analyses were performed to characterise the differences caused by *purL* alterations. Thirteen proteins were altered compared to wt. The corresponding genes were analysed by qRT-PCR not only in the Tn263 and wt, but also in clinical strains with different biofilm activity. Overall, this study suggests that *purL* is essential for biofilm formation in *E. coli* and can be considered as a potential antibiofilm target.

## 1. Introduction

*Escherichia coli* is a well-characterised microorganism frequently used as a laboratory model and in industrial microbiology [1]. In the human body, most gut-resident *E. coli* prevent colonisation by pathogenic bacteria and favour the host by producing vitamin K and B12, which are essential during the blood coagulation process and the formation of red blood cells, respectively [2,3,4]. However, some *E. coli* can also cause intestinal or extraintestinal infections such as urinary tract infections (UTIs), meningitis, and neonatal sepsis [5,6,7,8,9]. *E. coli* grows as free-living cells or biofilm-forming communities. Microbial biofilms are well-organized communities where microorganisms live embedded in a self-produced extracellular polymeric substance (EPS) which protects against adverse environmental conditions [6]. Biofilms are ubiquitous, being found attached to biological or inert surfaces in diverse ecological niches. In addition, *E. coli* biofilms cause several medical device-related infections, including joint infections, intravascular catheter infections, and catheter-associated urinary tract infections (CAUTI) [6,10,11]. Unfortunately, biofilm-forming organisms have an increased tolerance to antibiotics due to the reduced penetration of the antibiotic through the biofilm extracellular matrix (ECM) [12,13]. Likewise, the bacteria within the biofilms evade the immune system through various defence mechanisms, such as by avoiding the complement system and phagocytosis or by acting as a physical barrier [12]. Therefore, there is an unmet need to elucidate new ways to treat biofilm-related infections. In this sense, to understand the molecular mechanisms underlying biofilm formation, it is essential to develop efficient new clinical treatments. Several studies have described the mechanisms and gene regulation involved during the three general phases of biofilm formation: attachment, development, and dispersal [8,14,15,16,17]. For example, some of the well-characterised virulence factor genes among *E. coli* strains during the initial stages include type 1 fimbriae or pili, curli fimbriae, and flagella. Specifically, disruption in *fim* genes (*fimA* and *fimH*, that encode type-1 fimbrial protein A chain and type 1 fimbrin D-mannose specific adhesion, respectively) reduces initial attachment to abiotic surfaces and biofilm production [18]. Curli fimbriae, encoded by the *csgBAC* operon, provide initial adhesion to abiotic surfaces and promote cell aggregation [19]. Thus, alteration of the *csgA* gene triggers reduced biofilm formation due to a decreased production of the main curli protein CsgA subunit [20]. In addition, alterations in genes involved in motility (*fli*, *flh*, *mot*, and *che* alleles) also decrease biofilm formation [5]. On the other hand, the quorum sensing (QS) system plays a critical role in microbial biofilms during development and dispersal stages. 

Despite the current knowledge about these genes, the investigation of new essential genes/proteins during biofilm formation could elucidate unknown candidate genes with a higher potential as antibiofilm targets. In the present study we focus on protein expression and the comparison of gene transcription between an *E. coli* wt strain and the isogenic defective biofilm mutants (Tn) produced by transposon insertion. Taking this into account, the aim of this work was to identify new genes involved in biofilm formation in *E. coli* using phenotypic and molecular tools.

## 2. Materials and Methods 

### 2.1. Culture of E. coli and Electrocompetent Cells

The *E. coli* ATCC 25922 reference strain, used as the wt strain in this study, was grown for 24 h at 37 °C in Luria Bertani (LB) agar (Miller’s LB AGAR, Condalab, Madrid, Spain). Electrocompetent cells were made after the overnight culture of a single colony in LB broth (Miller’s LB Broth, Condalab) at 37 °C with shaking at 180 rpm. One millilitre of the overnight culture was used to inoculate 100 mL prewarmed (37 °C) LB broth and incubated in aerobic conditions at 37 °C under shaking at 180 rpm. When bacteria reached the mid-log phase (optical density (OD) at 600 nm = 0.6 ± 0.2), the cells were incubated on ice for 20 min. Bacterial cells were pelleted at 7000× *g* for 10 min, and washed three times: first with 50 mL of ice-cold water, second with 25 mL of ice-cold water, and lastly with 2.5 mL of ice-cold 10% glycerol. Electrocompetent cells were aliquoted and stored at −80 °C.

### 2.2. Transposon Mutagenesis 

Transposon mutagenesis was carried out according to the manufacturer’s recommendations (Lucigen). Briefly, 1 µL of EZ-Tn5™ <R6Kγori/KAN-2> Tnp Transposome™ kit (Epicentre Lucigen, bioNova Científica S.L., Madrid, Spain) was mixed with 45 µL of *E. coli* wt electrocompetent cells and included in a 0.2 cm electro-cuvette (Bio-Rad, Madrid, Spain). The cells were electroporated using the Pre-Set protocol from the Gene Pulser Xcell™ Electroporation Systems (Bio-Rad) at 25 µF, 200 Ohms, 2500 V. The electroporated cells were recovered immediately in 1 mL of Super Optimal broth with Catabolite repression (S.O.C.) medium (2% tryptone, 0.5% yeast extract, 10 mM NaCl, 2.5 mM KCl, 10 mM MgCl_2_, 10 mM MgSO_4_, and 20 mM glucose) and incubated at 37 °C with shaking (100 rpm) for 60 min. The incubated product was diluted 1:10, and 50 µL aliquots were plated onto LB agar with kanamycin-50 µg/mL. Each colony grown was stored separately at −80 °C in BD Difco™ Skim Milk (Becton Dickinson, Madrid, Spain) for further analysis. The efficiency of the electrocompetent cells was >10^7^ colony forming units (CFU)/µg of DNA.

### 2.3. Biofilm Analysis 

Five hundred and thirty-six of a total of 2578 transposon mutants were characterised in terms of biofilm production using a protocol previously standardised in our research group [21]. Briefly, all isolates were cultured overnight in LB agar at 37 °C in aerobic conditions. The cultures were established by the direct colony suspension method at 37 °C with shaking (180 rpm) in LB broth and then grown overnight. After incubation, colonies were diluted 1:100 in 200 µL of M63 medium (13.5 g/L KH_2_PO_4_, 2 g/L (NH_4_)_2_SO_4_, 5.0  ×  10^−4^ g/L FeSO_4_, 1 mL 1 M MgSO_4_·7H_2_O), supplemented with 0.25% glucose and adjusted to pH 7 (with KOH) and tested in 96-well flat-bottomed non-treated polystyrene microtiter plates with lids (Nunc™ Edge 2.0, VWR International, Barcelona, Spain) at 30 °C for 48 h. After incubation, biofilm was quantified using a crystal violet technique previously described by our group [21]. In brief, liquid culture from M63 medium was carefully removed, and the biofilm mass was washed twice with 210 µL of 1× phosphate-buffered saline (PBS) (pH 7.2) and dried at 65 °C for at least 20 min. Biofilms were stained with 200 µL of 2% (*v*/*v*) solution of crystal violet (CV) and incubated for 10 min at room temperature. Afterwards, the CV was completely removed, washed twice with 1× PBS and heat-fixed at 65 °C for 60 min. The CV was eluted by adding 200 µL of 33% acetic acid. Biofilm formation was measured at 580 nm using a Microplate Spectrophotometer (EPOCH 2 microplate reader, BioTek, Winooski, VT, USA).

The biofilm production of each sample was tested in triplicate, and samples showing an absorbance less than or equal to the positive control (ATCC 25922) were retested. Finally, the mutants were classified into three groups using the paired Student’s *t*-test (adjusted *p* < 0.05, considered significant and listed in Supplementary Table S1) comparing the normalised absorbance of each mutant (A Tn) with the absorbance of the reference strain (A ATCC). Only statistically different strains were considered to be LF or high biofilm formers (HF), whereas no statistically significant differences were considered as biofilm formers (F). 

### 2.4. Phenotypic Characterization of Mutants

#### 2.4.1. Growth Curves

To detect deficiencies in bacterial growth, the fitness of the selected mutants was tested. A single colony of each strain was grown overnight in LB broth at 37 °C with shaking at 180 rpm. After incubation, cultures were centrifuged and adjusted at OD_600nm_ = 1 with fresh LB, followed by dilution of 1:100 and plated onto 96-well flat-bottomed polystyrene microtiter plates with lids (Nunc™ Edge 2.0, VWR International, Barcelona, Spain). The plates were incubated at 37 °C with double orbital shaking (180 rpm). The absorbance was measured every 15 min for 24 h using the Epoch 2 Microplate Spectrophotometer. Each sample was repeated four times. LB without inoculum was used as a negative control. A plot of the natural log (Ln) of Absorbance versus time during the exponential phase yielded a straight line and the slope of this was equal to the specific growth rate (μ). The µ obtained for each strain was statistically compared to the μ of the wt strain.

Bacterial growth in M63 broth was measured by colony counting instead of Epoch 2, as the low growth of the mutant strain and the colourless medium limits its detection by spectrophotometer. Bacterial viability was also measured using the LIVE/DEAD^®^ BacLight Bacterial Viability Kit, which uses mixtures of SYTO^®^ 9 green-fluorescent nucleic acid stain and the red-fluorescent nucleic acid stain, propidium iodide (PI). According to the manufacturer, when both dyes are present, it is possible to distinguish between intact and damaged bacterial cells. PI penetrates only into bacteria with damaged membranes, causing a reduction in the SYTO^®^ 9 stain. Thus, bacteria with intact cell membranes stain fluorescent green, whereas bacteria with damaged membranes stain fluorescent red. Therefore, after incubation, biofilms were washed twice with 1× PBS (pH 7.2), cells were detached with a scraper, transferred into an Eppendorf tube, and sonicated at 37 kHz for 2 min using an ultrasonic bath (Fisher Scientific FB 15053, Waltham, MA, USA) following by vigorous vortexing for 1 min. The cells were then stained following the kit protocol and observed in the Olympus IX51 inverted fluorescence microscope. Three biological and technical replicates were done and three fields of each well were observed and imaged using the Fiji ImageJ software. Integrated density (staining intensity), defined as the sum of the values of the pixels in the image selected was measured and the percentage of viability was calculated. 

#### 2.4.2. Swimming Assay

A motility assay was performed as previously described [9]. Briefly, transposon mutant bacteria were incubated onto LB-kanamycin plates for 24 h at 37 °C. After incubation, a single colony was streaked in a 0.3% LB agar tube with 0.001% 2,3,5-triphenyl tetrazolium chloride (TTC). The tubes were incubated for 20 h at 37 °C. All completely red-stained tubes were considered positive. A motility assay was performed in triplicate for each strain. 

#### 2.4.3. Congo Red Assay 

Curli production was determined using the Congo Red (CR) assay as previously described by Prigent-Combaret et al. [20]. YESCA-CR agar plates were made with yeast extract and casamino acid agar (YESCA: 1 g L^−1^ yeast extract, 10 g L^−1^ casamino acids, 20 g L^−1^ agar), and autoclaved at 121 °C. After sterilisation, filter-sterilised CR and Brilliant Blue G (100 μg mL^−1^ and 10 μg mL^−1^ final concentrations, respectively) were added. Transposon mutants were streaked in LB broth and grown at 37 °C overnight. Five microliters of overnight culture were spotted on the centre of the CR agar plate and incubated at 28 °C for 48 h. Dark red colonies were indicative of adhesion fibers while white or light pink colonies were indicative that fibers were not produced.

#### 2.4.4. Hemagglutination Assays

Type 1 fimbriae (pili) can be tested by hemagglutination assays. The *fimH* gene encoded the mannose-specific adhesin, located at the end of the pilus, resulting in hemagglutination in the presence of mannose. Therefore, the presence of hemagglutination in the samples indicated the expression of FimH adhesin. The assay was performed according to the protocol described by Hultgren et al. [22]. Bacterial cells were grown overnight in LB agar. After incubation, each strain was suspended in 1× PBS to obtain an OD_600nm_ of 1.0. One mL of the suspension was centrifuged, and the pellet was resuspended in 0.1 mL of 1× PBS. Twenty-five µL (1 × 10^9^ to 2 × 10^9^ bacteria) were serially diluted in 96-well round-bottomed polystyrene microtiter plates containing 25 µL of 1× PBS in each well. An equal volume of 2.5% previously washed sheep erythrocytes was added and, after mixing, the plates were incubated at 4 °C for 4 to 18 h. The endpoint was defined as the highest dilution at which erythrocyte buttons were not observed. The titre was expressed as the reciprocal of the endpoint and then expressed in Log_2_. 

### 2.5. Genotypic Characterization of Mutants

#### 2.5.1. DNA Sequencing 

Two mutants with the most significant loss of ability to form biofilm were selected for DNA sequencing. Each strain was cultured onto LB agar plates at 37 °C in aerobic conditions for 24 h. One colony was inoculated in 5 mL of LB broth and placed on ice when the OD reached mid-log phase. DNA extraction was performed using the Bacterial Genomic DNA Isolation Kit (Norgen Biotek Corp, Schmon Pkwy, Thorold, ON, Canada) following the manufacturer’s recommendations. Then, DNA was quantified using a Qubit 4 Fluorometer (Thermo Scientific, Waltham, MA, USA). 

The genomic DNA material was used for the preparation of TrueSeq Illumina libraries (Illumina Inc, San Diego, CA, USA). The Illumina HiSeq2500 system was used as a sequencing platform with a 2× 125 bp paired-end strategy to generate 6,000,000 reads. The libraries were checked for quality analysis using FASTQC available at (http://www.bioinformatics.bbsrc.ac.uk/projects/fastqc/) implemented by the GPRO suit (https://gpro.biotechvana.com/).

The FASTQ files were pre-processed using Cutadapt software to remove primer adapters [23] and the sequencing quality was established with Prinseq with which reads with less than 50 bp and a concentration of up to 15% of Ns were discarded [24]. Then, de novo genome assemblies were conducted with the SPAdes Genome Assembler [25]. Reads were mapped onto the reference genome (*Escherichia coli*: GenBank: CP009072.1) using Bwa software [26]. The transposon was detected comparing the reconstructed genome to wt strain using the Mauve software [27].

#### 2.5.2. *purL* Gene Disruption

To confirm the role of *purL* in biofilm formation, the disruption of this gene was done using the λ red recombination system according to the protocol described by Datsenko and Wanner [28]. Briefly, the *E. coli* wt electrocompetent cells were electroporated with 50 ng of a red recombinase expression plasmid (pKD46). Transformants were grown in LB broth with ampicillin 100 µg/mL (Sigma-Aldrich, Milwaukee, WI, USA), and L-arabinose 10 mM (TCI Europe, Zwijndrecht, Belgium), at 30 °C to an OD_600_ of 0.6 ± 0.2 and then were remade as electrocompetent cells. 

The deletion of *purL* was obtained by PCR. To generate the PCR fragments, the primers were designed by priming, upstream and downstream, the sites flanking the chloramphenicol resistance gene in pKD3 and with ends homologous to upstream and downstream chromosomal sequences for targeting the *purL* gene. N-terminal deletion primer had a 50-nt 5’extension including the gene initiation codon and the 21-nt sequence 5’-GTGTAGGCTGGAGCTGCTTCG-3’. C-terminal deletion primers consisted of 21 nt for the C-terminal region including the termination codon, 29-nt downstream, and the 20-nt sequence 5’-CATATGAATATCCTCCTTAG-3’. The designed primers were: 5’-CGTTTCCCCCCCTTGGGTACACCGAAAGCTTAGAAGACGAGAGACTTATGGTGTA GGCTGGAGCTGCTTCG-3’ and 5’-CCGGGCTGCAATACCAATGGGTTGACGACTTACCCCAAC TGCTTACGTGCCATATGAATATCCTCCTTAG-3’ (Metabion). 

PCR products were gel-purified with the E.Z.N.A. ^®^ Gel Extraction Kit (Omega Bio-tek, Norcross, GA, USA) and digested with DpnI (Thermo Scientific™, Waltham, MA, USA). 500 ng of the re-purified product were mixed with 100 µL of electrocompetent cells carrying the pKD46 and electroporated. The cells were immediately added to 1 mL S.O.C. medium, incubated at 37 °C with shaking (180 rpm) for 2 h, centrifuged, and resuspended in 500 µL of fresh S.O.C. medium. Then, 250 µL were spread onto each LB agar plate containing chloramphenicol (Cm) 10 µg/mL to select Cm-resistant transformants. Afterwards, the obtained transformants were incubated at 43 °C in LB agar without antibiotics and tested for ampicillin sensitivity to test for loss of the pKD46 helper plasmid. Each colony was stored separately at −80 °C in BD Difco™ Skim Milk (Becton Dickinson, Madrid, Spain) for further analysis. Different PCRs were performed to demonstrate the successful disruption of the *purL* gene. The primers used are listed in Supplementary Table S2. 

The growth curve, biofilm formation, and curli fibers formation of the *ΔpurL::cat* knockout were also evaluated as previously described.

#### 2.5.3. *purL* Gene Complementation

The knockout strain was complemented to recover biofilm formation capacity. Briefly, purified PCR product of the *purL* gene was ligated to pGEM^®^-T vector System (Promega, Madison, WI, USA) using the T4 DNA Ligase and cloned into the competent knockout strain. The complemented bacteria were selected in LB agar plates containing ampicillin 100 µg/mL. Each colony was stored separately at −80 °C in Skim Milk for further analysis. Successful complementation was confirmed by PCR using the T7 promoter primer and an internal primer of the *purL* gene (Supplementary Table S2). Then, the biofilm formation, growth curves, and curli production tests of the complemented bacteria were assessed. 

#### 2.5.4. Effect of Inosine Addition to the Biofilm Formation Ability of the *purL* Mutant.

To examine the recovery of the ability to form biofilm of the *purL* mutant by the addition of an exogenous source of purine, M63 broth supplemented with double decreasing concentrations of inosine ranging between 50 and 1.56 µg/mL was used for biofilm formation analysis and growth curves, following the protocols previously described. Curli production was also measured by supplementing YESCA-CR agar with inosine 50 µg/mL.

### 2.6. Proteomic Characterization of Mutants

#### 2.6.1. Protein Isolation 

The protein isolation of the wt strain, as well as the proteomic isolation of the Tn263, the *E. coli* mutant selected for proteomic and transcriptional analysis, was performed as follows: the strains were cultured inoculating a single colony into LB broth for 18 h at 37 °C with shaking at 180 rpm to reach a cell concentration of 1 × 10^9^ CFU/mL. Colonies were diluted 1:100 in 70 mL of M63 medium supplemented with 0.25% glucose in a T75 non-treated flask (Thermo Scientific™ Nunc™, Waltham, MA, USA) and incubated at 30 °C for 72 h without shaking. The medium was carefully replaced by a fresh medium every 24 h. Subsequently, the medium was aspirated to remove planktonic cells, and flasks were washed twice with 20 mL of 1× PBS (pH 7.2) to remove unattached or loosely attached cells. Cells were then resuspended with 25 mL of 10 mM Tris-KCl (pH 7.8, 150 mM KCl) and sonicated at 37 kHz for 4 min using an ultrasonic bath (Fisher Scientific FB 15053, Waltham, MA, USA). Cells were harvested in 50 mL sterile falcon tubes (Deltalab, Rubí, Spain) and spun down at 3500× *g* for 10 min at 4 °C. The cell pellet was washed 3 times in 50mL of Tris-KCl and finally transferred into an Eppendorf tube and pelleted at 12,000× *g* for 10 min at 4 °C. The pellet was homogenized in lysis buffer (7M urea, 2M thiourea, 4% CHAPS, 2% ASB-14), and supplemented with protease inhibitor (GE Healthcare, Chicago, IL, USA). Bacterial cells were broken by sonication for 10 min with a cycle of 20 s on/59 s off at 4 °C on ice-water. After cell disruption, the samples were centrifuged at 3500× *g* for 15 min at 4 °C, and the supernatant was aliquoted and stored at −80 °C. The protein extracts were quantified using the 2-D Quant Kit and purified with the 2-D Clean-Up Kit (GE Healthcare). Fifty µg of protein was added to 0.5% of immobilized pH gradient buffer 4–7 and 20 mM of dithiothreitol (DTT). Each sample was assessed in three biological replicates. 

#### 2.6.2. Two-Dimensional SDS-PAGE Analysis

Two-dimensional sodium dodecyl sulfate polyacrylamide gel (2D SDS-PAGE) was performed as previously described [29]. Briefly, the Immobiline DryStrip Gels (IPG strips) (GE Healthcare) were hydrated following the manufacturer’s recommendations for 18 h at room temperature. The protein sample was loaded onto an IPG strip for first dimension separation by isoelectric focusing using 24 cm pH 4–7 linear IPG strips for 20 h (200 V for 2 h, 500 V for 3 h, 1000 V for 4 h, and 8000 V for 11 h, 20 °C) in an IPGphor I (GE Healthcare). After focusing, each strip was equilibrated in 10 mL of equilibration buffer (6M Urea, 75 mM Tris-HCl pH 8.8, 29.3% glycerol, 2% SDS and 0.002% bromophenol blue supplemented with DTT or iodoacetamide and 1% DTT) for 15 min and acetylated with iodoacetamide, and subsequently placed onto 12.5% polyacrylamide gels (24 cm × 20 cm) prepared as described by Laemmli [30]. The second dimension was performed using an Ettan^TM^ DALT six system (GE Healthcare). Gels were run at 20 °C using 80 V, 10 mA/strip, and 1 W/strip for 1 h, followed by 500 V, 40 mA/strip and13 W/strip until the bromophenol blue tracking front had run off the end of the gel.

#### 2.6.3. Gel Staining and Protein Detection 

Two-dimensional gels were silver stained as described previously [31]. Briefly, the gels were fixed for 18 h in 40% ethanol and 10% acetic acid, followed by incubation in sensitizing solution for 1 h in 30% ethanol with 0.02% (*w*/*v*) sodium thiosulfate. The gels were washed three times in distilled water for 5 min and treated with 0.1% (*w*/*v*) silver nitrate for 30 min. The gels were washed again twice with distilled water for 1 min. The developing step was performed with 3% (*w*/*v*) sodium carbonate and 0.025% (*v*/*v*) formaldehyde until the desired contrast was reached. Developing reaction was stopped with 1.5% (*w*/*v*) EDTA (Ethylenediaminetetraacetic acid)-Na_2_ for 45 min. Finally, the gels were washed twice with distilled water.

#### 2.6.4. Mass-Spectrometry Analysis for Orbitrap

Selected protein spots were excised from the gels digested with trypsin and analysed by liquid chromatography coupled to mass spectrometry (Orbitrap Velos, Thermo, Waltham, MA, USA), as described previously [32]. Proteins were identified through searching against *E. coli* proteins found in the UniProt database, using Mascot (Matrix Science, London, UK) to search the SwissProt database (2018_11, taxonomy restricted to *E. coli* proteins). Tandem mass spectrometry (MS/MS) spectra were sought with a precursor mass tolerance of 10 ppm, fragment tolerance of 0.05 Da, trypsin specificity with a maximum of two missed cleavages, cysteine carb-amido-methylation set as fixed modification, and methionine oxidation as variable modification. The significance threshold for the identifications was set at *p* < 0.05, with a minimum ions score of 20.

#### 2.6.5. RNA Isolation and cDNA Synthesis

*E. coli* ATCC 25922, the transposon mutant strain Tn263, as well as three biofilm-forming and three non-biofilm-forming clinical isolates were used for further gene expression analyses. Clinical strains belonging to a collection of our research group were collected over six months between 2016 and 2017 from different hospitals in Barcelona, Spain). All strains had been previously identified by MALDI-TOF mass spectrometry and their antimicrobial profile was tested according to the M100 guidelines (Clinical & Laboratory Standards Institute, CLSI 2019) [33]. Origins of isolation and antibiotic resistance profiles are shown in Supplementary Table S3. The strains were cultured by inoculating a single colony into LB broth for 18 h at 37 °C with shaking at 180 rpm to reach a cell concentration of 1 × 10^9^ CFU/mL. Colonies were diluted 1:100 in 70 mL of M63 medium supplemented with 0.25% glucose in a T75 flask for 72 h and 30 °C without shaking. Subsequently, the medium was aspirated to remove planktonic cells, and flasks were washed twice with 20 mL of sterilized 1× PBS (pH 7.2). Cells were resuspended in 3 mL of 1× PBS and treated with 6 mL of RNAprotect Bacteria Reagent (Qiagen, Hilden, Germany) and vortexed and centrifuged at 5000 rpm for 20 min at room temperature. The supernatants were discarded, and the pellets were treated with TE-lysozyme buffer (3 mg/mL in TE buffer pH 7.4). Afterwards, RNA was extracted using the RNeasy Mini Kit (Qiagen) according to the manufacturer’s recommendations. The total RNA was eluted in 50 µL of RNase-free water. RNA was isolated from four independent samples.

The extracted RNA was treated with DNA-free™ Kit DNase Treatment and Removal Reagents (Ambion, by Life Technologies AM1906, Waltham, MA, USA) to remove DNA contamination. After DNase treatment, RNA was tested for residual genomic DNA contamination by polymerase chain reaction (PCR) amplification of the *adk* gene and gel electrophoresis. 

The total RNA concentration was quantified using NanoDrop 1000 (Thermo Scientific, Waltham, MA, USA). An A260/280 ratio between 1.80 and 2.10 was considered optimal for RNA quality. cDNA was synthesized using 500 ng of RNA in a reaction volume of 20 µL and the qScript cDNA SuperMix reagent (Quanta Bioscience, 95048-100, Gaithersburg, MD, USA). The PCR amplification protocol consists of one incubation step at 25 °C for 5 min, a DNA polymerization step at 42 °C for 30 min, and a final enzyme deactivation step at 85 °C for 5 min. The final PCR product was diluted 1:10 and stored at −20 °C. cDNA was used as a template for quantitative reverse transcription PCR (qRT–PCR) assays.

#### 2.6.6. Gene Expression by qRT-PCR

qRT-PCR was carried out using 10 µL of PowerUp^TM^ SYBR Green Master Mix (Applied Biosystems, Thermo Fisher Scientific, A25742, Foster City, CA, USA), 1 µL of primer mix (each reverse and forward primers at a final concentration of 0.5 µM), 4µL of RNase-free water and 5µL of cDNA template. 

Reactions were conducted in duplicate and the *arcA* gene was used as the reference gene for normalisation of CT-values [34,35]. A non-template control reaction mixture was included for each gene. Amplification was performed using StepOnePlus^TM^ Real-Time PCR Systems (Applied Biosystems, Foster City, CA, USA) being the cycling conditions: a pre-incubation step of 95 °C at 15 min, followed by an amplification step of 95 °C for 10 s, 60 °C for 30 s and 72 °C for 30 s repeated for 44 cycles, and a melting curve analysis from 60 °C to 95 °C in 0.3 °C intervals. The relative fold-change of mRNA transcripts in biofilms compared to mutant cultures was calculated using the 2^−ΔΔCt^ method. The primers used are listed in Supplementary Table S4. The primers designed in this study were made using Primer Express Software v.3.0.1 (Applied Biosystems). Data analysis was based on at least three independent experiments.

### 2.7. Data Plotting and Statistical Analysis

All statistical analyses were performed using GraphPad Prism v8.0.2 software (La Jolla, CA, USA) unless otherwise stated. Graphs were created using GraphPad Prism v8.0.2 software and Tableau Software (Seattle, WA, USA). Growth rates were evaluated via two-tailed Student *t*-test. One-way ANOVA followed by post hoc Dunnett’s multiple comparison tests were used to analyse colony counting in M63, LIVE/DEAD assay, and biofilm quantification after the addition of inosine. Statistical quantification of relative protein levels was analysed with Progenesis SameSpots 4.6.206 (Totallab, Newcastle, UK) and evaluated via ANOVA. The relative transcript levels of selected genes in wt strain, Tn263 mutant and clinical isolates were calculated using the 2^−ΔΔCt^ method. Two-tailed student *t*-test was performed to compare wt vs Tn263 and biofilm-former vs non-biofilm former Differences were considered statistically significant with a *p* value < 0.05. 

## 3. Results

### 3.1. Mutant Selection by Biofilm Assays 

**Figure 1:**
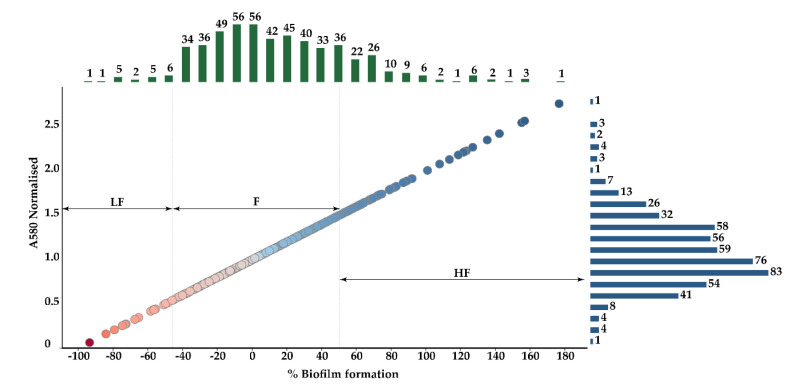
Scatter plot and marginal histograms of the mutants. Upper bars (green) indicate the number of strains in the respective percentage of biofilm formation. Right bars (blue) indicate the number of stains in the respective absorbance values. Each spot represents a mutant where red colour indicates low biofilm formation rates and blue corresponds to high biofilm formation rates. LF: low biofilm former; F: biofilm former; HF: high biofilm former; A: absorbance.

A total of 2578 *E. coli* mutants by transposon insertion were generated, of which 536 were analysed in this study. The mutants were further studied for their ability to form biofilm and compared to the wt strain. Mutants presenting reduced biofilm-formation capacity (Table S1) were selected. A total of 116 mutants showed an altered biofilm formation rate in comparison with the wt strain. Among these, 20 were classified into the low biofilm formers (LF) group (3.73%) with an absorbance between 0.065 and 0.584, 420 mutants classified as formers (F) (78.36%) with an absorbance from 0.588 to 1.414 and finally 96 were classified into the high biofilm formers (HF) (17.91%) with an absorbance higher than 1.414 (Figure 1). Into the low biofilm formers, Tn263 and Tn463 mutants showed the most significant loss of biofilm formation with values of absorbance of 0.065 and 0.159, respectively.

### 3.2. Phenotypic Characterization of Mutants 

The 20 mutants classified into the low biofilm-forming capacity group (LF) were assessed with a phenotypic comparative study.

#### 3.2.1. Growth Curves in LB Broth

**Figure 2:**
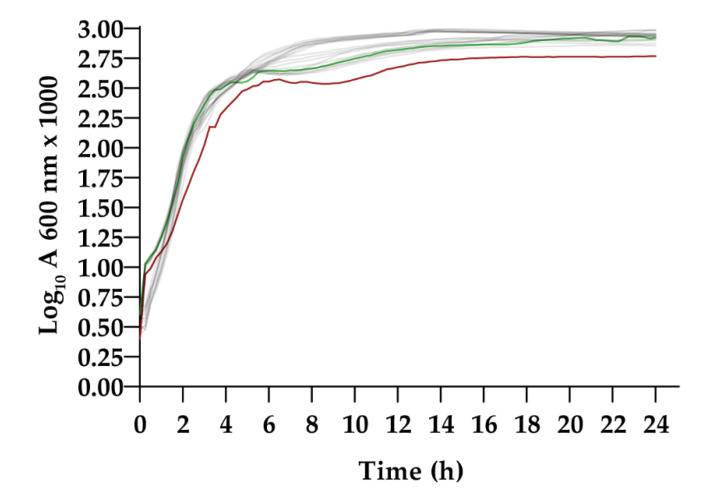
Fitness assay in Luria Bertani (LB) broth. Green represents the wild type curve; Red represents Tn463. Grey represents the other 19 low biofilm-former (LF) transposon mutants, including Tn263. Growth rates were statistically evaluated via two-tailed Student *t*-test.

Transposon insertion can affect essential metabolic pathways and alter normal physiological development. To detect these possible defects, growth curves were evaluated on 20 LF mutant strains and compared to the wt strain (Figure 2). The analysis of the specific growth rate (μ) performed in LB broth showed statistically significant differences in the Tn463 mutant in comparison to the wt (*p* < 0.0001). The specific growth rates (μ) of the other mutant strains were similar to the wt, and no statistically significant differences were found (*p* > 0.05). Figure 2 shows the entire growth curve measuring absorbance every 15 min until 24 h of incubation.

#### 3.2.2. Swimming, Congo Red and Hemagglutination Assays

In this section, 20 mutants were examined according to the phenotypic characterization of non-biofilm-forming mutants. The results of the swimming, curli fibers production and hemagglutination assays are summarized in Table 1. 

In the swimming assay, no samples were found to be non-motile, indicating that defects in biofilm formation are not related to defective flagella. Supplementary Figure S1 shows an example of swimming classification using a defective mutant in the *flhD* gene as non-motile control and wt as motile control. The ability to form curli fimbriae was evaluated using the CR assay in YESCA-CR plates. In this assay, the Tn463 and Tn263 mutants showed significant differences caused by altered or defective synthesis of curli. The results obtained in this assay are further discussed in Section 3.3.2. Finally, all selected mutants showed positive hemagglutination, suggesting that type 1 pili are not involved in the lack of biofilm formation in these mutants. Since Tn463 and Tn263 showed the the most significant loss of biofilm formation and some changes in these assays, we analysed only these mutants in the next section.

### 3.3. Genotypic Characterization of Mutants

#### 3.3.1. DNA Sequencing 

Since this study focused on low-forming strains after transposon insertion, the Tn263 and Tn463 mutants were selected for DNA sequencing. Although this was not within the scope of the present study, the characterisation of the mutants with increased biofilm production could also lead to interesting results and warrants further investigations.

**Figure 3:**
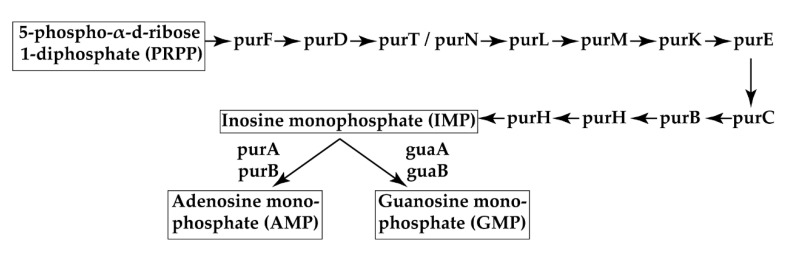
De novo purine biosynthesis in *E. coli.* Adapted from Kyoto Encyclopedia of Genes and Genomes (KEGG) pathway maps.

According to the analysis of transposon insertion realised in collaboration with BIOTECHVANA (Valencia, Spain), in the Tn463 mutant, the transposon was inserted in the *csgA* and *cysB* genes. The *csgBAC* operon encodes the curli major subunit protein CsgA, the curli nucleator protein CsgB, and the periplasmic chaperone CsgC [36]. The importance of curli fibers in biofilm formation has previously been demonstrated as being linked to the synthesis of the exopolysaccharide cellulose, a complementary factor of the biofilm matrix [37]. *cysB* gene encodes for CysB protein which is a positive regulator of gene expression for the cysteine regulon, a system of ten or more loci involved in the biosynthesis of L-cysteine from inorganic sulfate. Since *csgA* is a gene that has been previously related to biofilm formation, we focused only on Tn263 for further analysis. In Tn263, the transposon was only inserted in the *purL* gene. *purL* encodes for phosphoribosyl-formyl-glycinamidine synthase, involved in the de novo purine biosynthesis pathway using 5-phospho-α-d-ribose 1-diphosphate (PRPP) as a precursor for producing inosine monophosphate (IMP), which is the precursor of adenosine monophosphate (AMP) and guanine monophosphate (GMP) (Figure 3). Interestingly, in our mutant, the transposon insertion is located after 134 bp, position 45 in the protein affecting the N-terminal domain and producing an unstable and non-functional protein but not affecting the gene expression. Therefore, purine synthesis appears as an essential function whereby bacteria produce nucleotides for the synthesis of DNA and RNA. Given the impact on the purine biosynthesis pathway, other metabolism alterations in Tn263 were investigated using 2D SDS-PAGE analysis and subsequent protein identification with orbitrap.

#### 3.3.2. Confirmation of the Role of the *purL* Gene in Biofilm Formation Using Its Knockout Strain 

The *ΔpurL::cat* (Cm^R^), Tn263, and wt strains were cultivated in M63 broth. The mean value of colony-forming units showed statistically significant differences in biofilm as well as planktonic culture in both *ΔpurL::cat* and the Tn263 mutant strains compared to the wt strain after 72 h of incubation in M63 broth. In the same line, the measure of integrated density showed significant differences in the total number of cells in biofilm (*p* = 0.0002 and *p* = 0.0008, Tn263 and *purL* mutant, respectively). Nonetheless, the percentages of live and dead bacteria were highly similar between them (Table 2).

**Figure 4:**
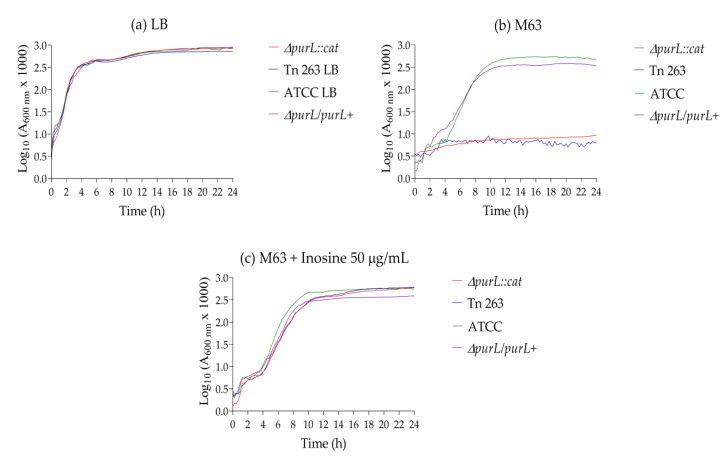
Fitness assay. (**a**) Growth curves in LB broth (**b**) Growth curves in M63 broth. (**c**) Growth curves in M63 broth supplemented with inosine (50 µg/mL). Growth rates were statistically evaluated via two-tailed Student *t*-test.

However, similar growth curves of the *ΔpurL::cat* and the Tn263 mutant strains compared to the wt strain were observed when the M63 culture media was supplemented with inosine (50 µg/mL). The complemented strain *ΔpurL/purL+* showed similar growth curves to the wt strain in LB as well as in M63. Supplementation with inosine did not have a significant effect on the growth curve of the complemented strain. It should be noted that the general growth in the supplemented M63 broth shows differences in the Log phase compared to the Log phase obtained with LB broth (Figure 4).

**Figure 5:**
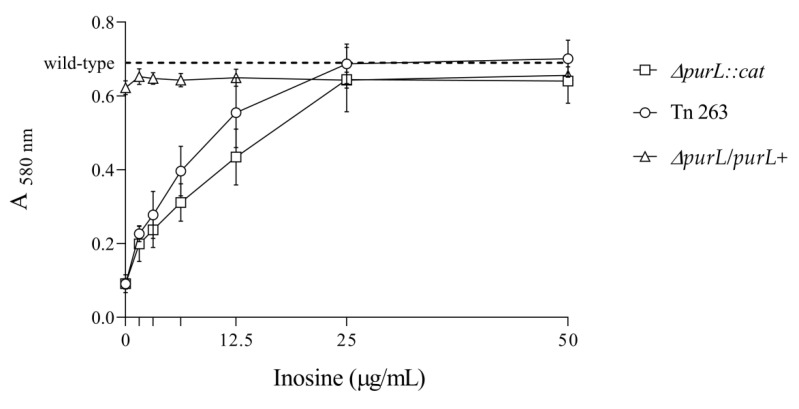
Biofilm formation of *ΔpurL::cat* mutant, Tn263, and the complemented strain *ΔpurL/purL+* using different concentrations of inosine. Each point in the curve represents mean values of A_580nm_ after 48 h of incubation; vertical bars correspond to standard deviations. The dashed line shows the absorbance value of the wild type strain, used as control. The results were analysed by One-way ANOVAs followed by post hoc Dunnett’s multiple comparisons tests.

As already observed with the Tn263, the *ΔpurL::cat* mutant was not able to form biofilm in M63 broth. However, when the medium was supplemented with inosine (50 µg/mL), both the *ΔpurL::cat* and the Tn263 mutants recovered the ability to form biofilm. Likewise, no statistically significant differences after One-way ANOVAs followed by post hoc Dunnett’s multiple comparisons tests were found using inosine concentrations ranging between 25 and 50 µg/mL for the *ΔpurL::cat* and the Tn263 mutants compared to the wt (*p* > 0.05). Nevertheless, inosine concentrations below 25 µg/mL were insufficient to recover the ability to form a biofilm by the *purL* affected strains (Figure 5). Then, after complementation with the *purL* gene using the pGEM^®^-T vector, the knockout strain also recovered the ability to form biofilm, and the addition of inosine did not have a significant effect on its biofilm formation. The complemented strain *ΔpurL/purL+* shows similar absorbance values than the wt strain. The addition of inosine does not have a significant effect on its ability to form biofilm.

**Figure 6:**
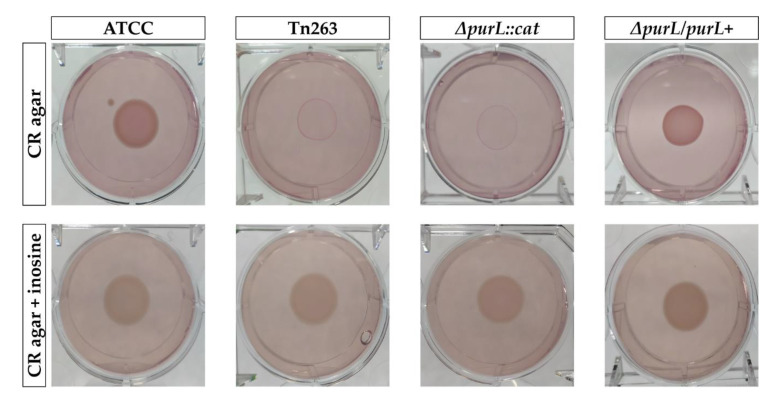
Curli production. Dark red colony in yeast and casamino acid agar (YESCA-CR AGAR) (ATCC and *ΔpurL/purL+*) represents a curli producer, and light pink colonies (Tn263 and *ΔpurL::cat*) were associated with the defective phenotype. The defective phenotype of both mutants was restored in the YESCA-CR + Inosine agar.

Curli production was also measured by CR assay. *ΔpurL::cat* and Tn263 mutants showed an inability to bind CR and produced less curli fibers compared to the wt and the complemented strains. Curli production was restored in the mutants by supplementing agar medium with inosine 50 µg/mL, demonstrating the interplay between curli production, purine biosynthesis pathway, and biofilm formation (Figure 6).

### 3.4. Proteomic Characterization of Mutant Tn 263

#### 3.4.1. Comparative Proteomic Analyses of the wt and Tn263 by Two-Dimensional SDS-PAGE

**Figure 7:**
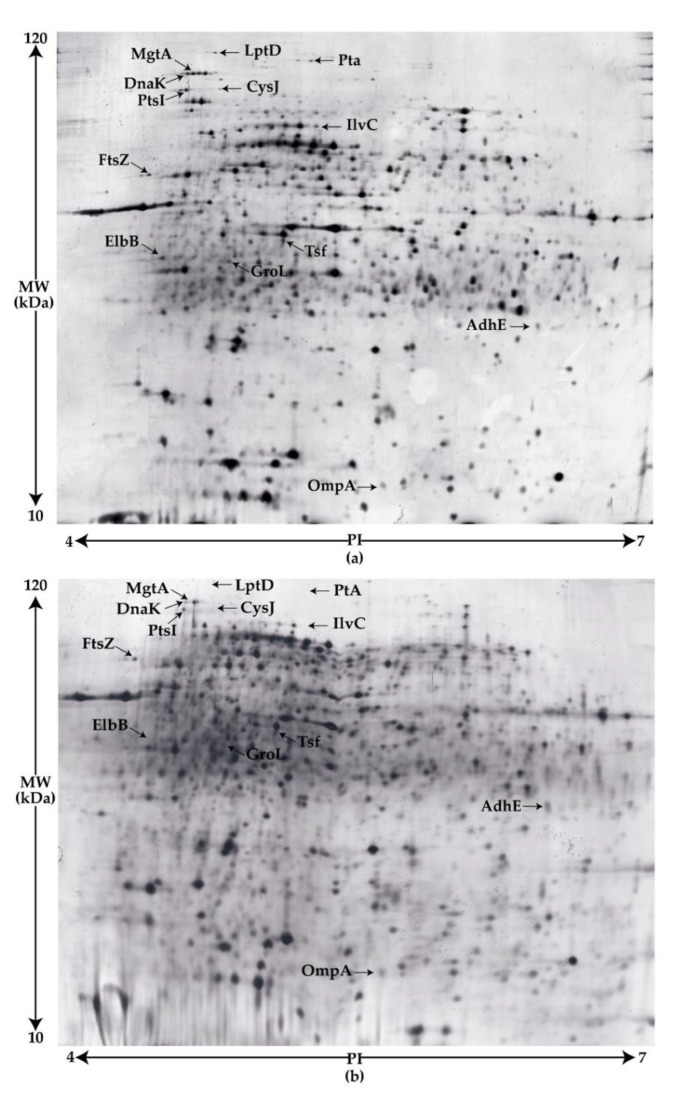
Two-dimensional sodium dodecyl sulfate polyacrylamide gel (SDS-PAGE) images. (**a**) wt strain; (**b**) Tn263. The arrows mark significant differences (ANOVA *p* < 0.05) among the strains detected by Progenesis SameSpots 4.6.206. Further information about each spot identified can be found in the supporting information (Supplementary Table S5).

To investigate the effect of transposon insertion at a molecular level, the Tn263 mutant was compared to the wt strain using two-dimensional SDS-PAGE and mass spectrometry. Three biological replicates of each sample were studied (Figure 7, Supplementary Figure S2, and Supplementary Figure S3). Thirteen statistically significant spots were identified, 12 present in the wt strain, and one in the mutant strain. Among these, only spots with a score over 25 were considered for posterior analyses (ANOVA *p* < 0.05) (Supplementary Table S5). The molecular weight (Mw) and isoelectric point (pI) of proteins were determined using MASCOT software and are in line with their theoretical Mw/pI. The higher Mw might be the result of covalent binding, although lower Mw may be produced by proteolysis or post-translational changes. Figure 7 provides a representative image of 2D-gels in which spot differences are highlighted with arrows.

After mass-spectrometry identification and following pathway analysis, 13 proteins were classified into three categories: metabolism and cell maintenance; genetic information processing and signalling and cellular processes.

Within the metabolism regulation six proteins were included: CysJ, IlvC, ElbB, MgtA, Pta and AdhE. CysJ is a nicotinamide adenine dinucleotide phosphate (NADPH) flavin oxidoreductase that participates in the sulfite reductase complex (CysJ8I4). This protein is encoded by the *cysJ* gene, which plays an essential role in sulphur metabolism, required for de novo biosynthesis of L-cysteine. Aside from biosynthesis pathways, *cysJ* is also related to the detoxification system of toxic and mutagenic N-hydroxylated nucleobases [38]. In addition, IlvC, a Ketol-acid reducto-isomerase encoded by the *ilvC* gene, is seen to participate during valine, leucine, and isoleucine biosynthesis. Glyoxalases are principally involved in the conversion of glyoxal to glycolate, therefore facilitating the conversion of toxins to a non-hazardous product [39]. *E. coli* possesses four glyoxalases which belong to the DJ-1 superfamily, i.e., HchA, YajL, YhbO, and ElbB, the latter present in our analysis. On the other hand, MgtA, a magnesium-transporting adenosine triphosphatase (ATPase), has a crucial function in transmembrane transport of electrons or protons. Phosphate acetyltransferase encoded by the *pta* gene is the first enzyme of the acetate pathway in the aerobic metabolism. The enzyme catalyzes the interconversion of acetyl-phosphate and acetyl coenzyme A. Finally, the only protein overexpressed in the Tn263 mutant was an aldehyde-alcohol dehydrogenase, encoded by the *adhE* gene (*p* = 0.0025). Under anaerobic conditions, AdhE catalyzes the reduction of acetyl-CoA to acetaldehyde, leading to an increase of NAD^+^.

Regarding the proteins identified within the genetic information processing group, we identified the elongation factor thermostable (EF-ts) which is associated with the elongation factor Thermo-unstable (EF-Tu) and regulates the exchange of Guanosine 5’-Diphosphate (GDP) to GTP via aminoacyl-tRNA. The union of EF-ts to ternary structures (EF-Tu-GTP-aa-tRNA) could increase the maximum rate of translation [40]. The cell division protein FtsZ regulated by GTP hydrolysis was also detected. This protein marks the site where the Z-ring structure divides the cell. Z-ring mutation could alter the structure and lead to both the development of functional and non-functional cells [41]. Included in the list there was also the DnaK chaperone, the dominant bacterial heat shock protein (Hsp 70). This is involved in protein secretion and chromosomal DNA replication and interacts with σ^32^ for controlling heat shock response. The last protein was the 60 kDa chaperonin GroL. The GroL chaperonin, also stimulated by σ^32^, mediates the assembly of unfolded polypeptides generated under stress conditions, and together with its regulator GroES, it is necessary for the proper folding of specific proteins.

Last, within the signalling and cellular process, we found the LptD (lipopolysaccharide transport) protein which, in combination with LptE protein, has an essential role in the production of lipopolysaccharide (LPS), the predominant component at the surface of the outer membrane (OM) of all Gram-negative bacteria. We also found the phosphoenolpyruvate-protein phosphotransferase enzyme 1 (PtsI) which participates in the phosphotransferase system related to phosphorylation of carbohydrates in *E. coli*. Finally, outer membrane protein A (OmpA) was identified as an abundant protein in *E. coli*, and it plays a significant role in maintaining cell integrity [42].

#### 3.4.2. Verification of Biofilm-Related Genes by q-RT-PCR 

The protein coding-genes corresponding to the proteins previously identified by SDS-PAGE and mass spectrometry were analysed to investigate possible translational alterations in the mutant sample. Further validation of these interesting targets was conducted by qRT-PCR between Tn263, the wt strain and the three biofilm-formers and three non-biofilm-formers clinical isolates.

**Figure 8:**
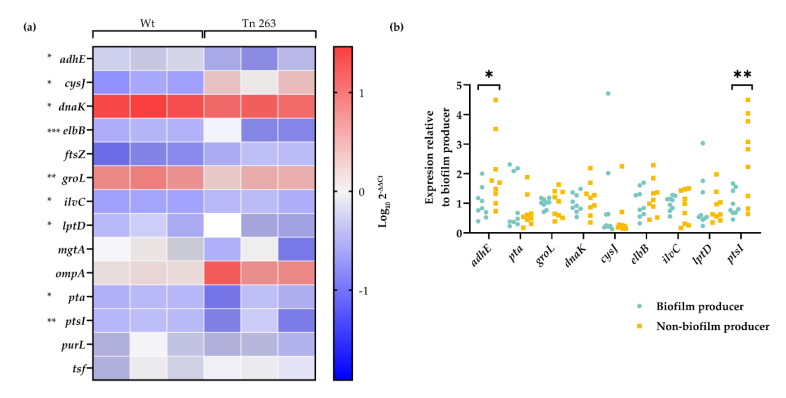
Analysis of the expression of selected genes in the wt and Tn263 mutants and in clinical isolates. (**a**) Heatmap showing the expression levels of biofilm-related genes in the wt and Tn263 mutant strains. Expression is presented as a logarithm of their 2^−ΔΔCt^. Each square shows a biological replicate (n = 3 vs 3). (**b**) Scatter plot showing the expression of selected genes in biofilm producer and non-biofilm producer clinical isolates. Expression is normalised relative to the average expression of the biofilm producer isolates per each gene (n = X vs X). Each clinical isolate was performed in triplicate. T test: * *p*< 0.05, ** *p* < 0.01, *** *p* < 0.001.

mRNA detection by qRT-PCR and the subsequent analysis by 2^−ΔΔCt^ method showed that three genes were downregulated (*dnaK*, *groL*, and *adhE*) and six genes were overexpressed (*lptD*, *cysJ*, *pta*, *ilvC*, *elbB* and *ptsI*) in the Tn263 mutant compared to the wt strain. Four genes did not present changes among groups (*tsf*, *ftsZ*, *ompA*, and *purL*) (Figure 8a). To investigate whether these expression changes were related to the decreased biofilm formation capacity of the mutant, we assessed the expression levels of these genes in a collection of well-characterized *E. coli* clinical isolates. Two genes, *adhE* and *ptsI* presented a significant upregulation in the non-biofilm producers compared to the biofilm producer strains (*p* = 0.0347 and *p* = 0.0169, respectively) (Figure 8b). In the first case, the Tn263 mutant showed upregulation of the AdhE protein, while it showed a low mRNA expression. However, mRNA expression in non-biofilm-formers remained upregulated. In the last case, the production of the PtsI protein was detected as being upregulated in the wt strain, whereas mRNA transcriptional levels were upregulated in both, the Tn263 and non-biofilm-formers.

## 4. Discussion

Bacteria have largely been investigated as planktonic cells. However, in many cases, bacteria grow in communities known as biofilms. These structures offer evolutionary advantages over their competitors and enable them to overcome environmental stresses. It is therefore important to understand the factors involved in the development of microbial biofilms that give them phenotypes different from their planktonic counterparts.

“Omics” techniques, such as high-throughput DNA sequencing, transcriptomic approaches, or proteomics, can clarify the underlying mechanisms of biofilm production. Progress in this field has indeed led to numerous studies that provide insight into molecular approaches [43]. 

The present study aimed to determine new genes involved in biofilm formation in *E. coli* using phenotypic and molecular tools comparing *E. coli* wt strain and the isogenic defective-biofilm mutants (Tn) generated by transposon insertion. Although the characterisation of the mutants with increased biofilm production was not within the scope of the present study, this could also lead to interesting results and warrants further investigations.

In the transposon mutant library, we found deficient mutants in biofilm formation. Two of these with the most significant loss of biofilm formation capacity were selected for the following analyses. 

Tn463 was initially selected as a non-biofilm-forming mutant in the first phenotypic assays. However, it was later discarded because it had altered fitness and expression in curli fimbriae compared to the wt strain. Besides, the transposon was inserted into the *csgA* gene, a previously described gene that is involved in biofilm formation. 

The second one, Tn263 mutant, differed from the wt in its proteomic and transcriptional profiles. DNA sequencing revealed a transposon insertion in the *purL* gene, which is involved in the de novo purine biosynthesis pathway. This pathway plays a critical role in the synthesis of DNA and RNA. In *E. coli*, the structural gene *pur* has various loci either individually (*purT, purL, purC, purA*) or collectively within operons (*purF, purHD, purMN, purEK*) [44]. Concretely, *purL gene* codes for formyl-glycinamide ribonucleotide amido-transferase (FGAR-AT), which is an enzyme that catalyses the fourth step of the purine biosynthetic pathway. This is an ATP-dependent conversion of formyl-glycinamide ribonucleotide (FGAR) and glutamine to yield N-formylglycinamidine ribonucleotide (FGAM), adenosine diphosphate (ADP), Pi, and glutamate. In our mutant, the transposon was located in the N-terminal domain of the *purL* gene. Previous studies related three principal domains in *purL:* the glutaminase, the FGAM synthetase, and the N-terminal domains [45]. The glutaminase domain produces ammonia, which is transported to the FGAM synthetase domain. The N-terminal domain possesses an essential function for ammonia channel formation and posterior union between the two catalytic domains [46]. Changes in the N-terminal domain could potentially alter the interactions between other structures and block their function without transcriptional modification. 

In order to determine whether the *purL* mutation could cause a fitness deficiency that would impede the biofilm formation, the mutant strain was grown in LB, without changing its growth rates, which suggests that the *purL* mutation does not affect its fitness in this growth medium. However, fitness was altered in M63 due to the alteration in the purine biosynthetic pathway. Biofilm formation was carried out in M63 medium at 30 °C to favour the expression of genes involved in the biofilm formation [20,21,47,48,49,50] as curli fimbriae, aggregated amyloid structures which promote cell aggregation and attachment to abiotic surfaces. They are promoted at a temperature less than 32 °C inducing transcription of the *csgDEFG* operon [51]. Nonetheless, Tn263 and *ΔpurL::cat* mutants showed poor biofilm formation in the M63 medium compared to the wt strain. On the other hand, when we used the M63 broth supplemented with inosine in different concentrations, both mutants, Tn263 and *ΔpurL::cat*, regained the ability to form biofilm. Since both strains can form biofilm in the inosine-supplemented M63 medium, the lack of biofilm formation could be attributed to the *purL* disruption. The result was confirmed by the complementation of the knockout strain. Thus the lack of purines tends to reduce RNA synthesis by RNA polymerase or amino acid starvation resulting in a deficient biofilm phenotype. Nhu et al. found that the de novo purine biosynthesis is a critical pathway in curli production [36]. They hypothesise that a disruption in this pathway reduces the cyclic-di-GMP concentration inside the cell triggering the transcription of the master regulator *csgD*. The authors found that *purF, purD, purM* and *purK* mutants were unable to bind CR on YESCA-CR agar. In the same line, Garavaglia et al. also found that the mutation of the purine biosynthetic gene *purH* resulted in the inability to produce curli fibers [52].

Our results support these previous findings because the *ΔpurL::cat* mutant exhibits a curli-deficient phenotype, resulting in decreased biofilm. However, after supplementation of the growth medium with inosine, both mutants, Tn263 and *ΔpurL::cat*, restored curli assembly suggesting that *purL* alteration and subsequent dysfunction in the purine pathway hinder the efficient transcription of the *csgDEFG* operon and *csgD* expression [52].

Biofilms are an important virulence factor involved in surface colonisation and subsequent infection, often causing chronic infections. A change in the purine synthesis pathway could reduce the colonisation rate and thus decrease biofilm infection. For example, disruption in the *cvpA-purF* or only the *purF* genes related to the de novo purine biosynthesis reduces uropathogenic *E. coli* (UPEC) internalization into bladder epithelial cells without alteration in epithelium colonisation [53]. Along the same lines, one study demonstrated that *Burkholderia*, defected in *purl* was unable to establish symbiotic accommodation in *Riptortus* sp. *Burkholderia* did not colonize *Riptortus* sp, even though the bacteria were capable of colonizing the host midgut. These results suggested that purine biosynthesis also could play an essential role in infection.

After finding that transposon was inserted into the *purL* gene, additional studies were conducted on the changes that this insertion caused at proteomic and transcriptional levels between wt and Tn263. 

Comparative proteome analysis revealed 13 proteins statistically significant among wt and Tn263 (Figure 7). Among these proteins, five were found to be closely associated with biofilm regulation. The two chaperones, DnaK and GroL, are involved in stress response that usually results in the passage from individual cells to biofilm, inducing the loss of the flagella and performing maintenance activity in mature biofilm. Specifically, DnaK was involved in the transcriptional regulation of *flhDC*, responsible for the flagellum biogenesis, an important organelle associated with biofilm formation, adhesion, and colonisation [54]. Moreover, a recent work by Sugimoto et al. [55] showed that the DnaK protein contributes to curli formation. Via *rpoS*, DnaK modulates the expression of the *csgDEF* operon which encodes for CsgD (master transcriptional regulator of curli) acting as a positive regulator of the *csgBAC* operon. This last operon encodes the CsgA and CsgB units, the major and minor structural components, respectively. On the other hand, studies in *Cutibacterium acnes* hypothesized that the GroL protein might contribute to the organisation of the biofilm matrix given its ability to bind to external DNA [56]. For this reason, the detection of proteins involved in maintaining a mature biofilm maintenance in the wt strain supports the idea that the Tn263 mutant was unable to form a biofilm.

Pta enzyme could be indirectly modulate the biofilm formation through acetate intermediates that can activate regulatory cascades of biofilm formation [57]. For example, acetylation produced by acetyl-coA from CpxA and UvrY, both members of the two-component regulatory system or RcsB, are involved in numerous regulatory DNA regions such as colanic acid synthesis, which enhances the 3D structure of the biofilm [58,59,60].

Another protein involved in biofilm formation that was detected in wt strains was PtsI. In this case, previous studies on *Bacillus cereus* showed that mutations in the *ptsI* gene cause a 70% decrease in biofilm rates [61]. In avian pathogenic *E. coli* (APEC), *ptsI* deletion resulted in motility and biofilm-deficient bacteria, among other pathogenesis and phenotypic changes [62]. Furthermore, in *E. coli*, the internalisation of AI-2, a QS regulator, via Lsr activation, is mediated through *ptsI* phosphorylation [63]. Our results reinforce the hypothesis that PtsI is synthesized when bacteria develop the biofilm, given that PtsI is used to internalise AI-2 in the matrix. The fact that the mutant strain synthesises a lower amount of PtsI indicates that it is not in confluence and, therefore, it does not need to synthesize the protein. The last protein detected at high rates and associated with biofilm regulation in wt strain was OmpA. Indeed, the study by González Barrios et al. showed that the presence of an isogenic OmpA mutant decreases biofilm formation [64]. 

Finally, the only protein with high rates in Tn263 was AdhE. We hypothesise that the mutation in the *purL* gene leads to a hypoxic state, therefore activating anaerobic respiration. This idea is reinforced by studies carried out by Leonardo et al. who showed that using glucose as a carbon source in anaerobic conditions decreases NADH/NAD^+^ ratio and changes the expression of the *adhE* gene [65]. Hence, the *adhE* gene would be regulated at the transcriptional level by the NADH/NAD^+^ ratio. 

Colón-González et al. also explained that anaerobic growth does not support biofilm formation in *Escherichia coli* K-12 by the enzymatic activity of the AdhE protein. *adhE* gene is induced during oxygen deficiency, and the authors found significant differences of growth in Congo red agar, where aerobically grown cells were red (curli +) and anaerobically grown cells were white (curli −) [66]. This suggests a possible interaction between AdhE expression and defective curli fibers under anaerobic conditions. AdhE was found to be upregulated in Tn263 compared to the wt strain and does not produce curli fibers or biofilm. However, this gene is not upregulated among non-biofilm forming clinical strains. Therefore, we hypothesise that the expression of AdhE in this mutant could be strain-dependent because it has the *purL* gene affected by the effect of transposon insertion, whereas the *purL* gene is not affected in the wt or the clinical strains. 

In summary, we have found a downregulation of Tn263 in the above mentioned proteins (with the exception of AdhE), all of which are related to the development and maintenance of the biofilm. Presumably, stress conditions caused by purine deficiency led to DNA damage in Tn263, which reduced its translational activity.

At the transcriptional level, the corresponding genes of these 13 proteins were analysed revealing interesting observations. The levels of expression of *lptD, pta, elbB, ptsI*, *cysJ* and *ilvC* genes were higher in the Tn263 compared to the wt strain. Interestingly, *elbB* gene was overexpressed significantly in the Tn263 strain (*p* < 0.001). This overexpression can be caused by a high level of oxidative stress, which increases the production of glyoxals [67]. The last gene related to metabolic pathways, *pta*, showed increased transcriptional levels in the mutant strain, suggesting that it could use anaerobic metabolic pathways to synthesize ATP. Transcriptional changes related to the genetic information processing group showed lower levels of *dnaK* (*p* < 0.05) and *groL* (*p* < 0.01) in the Tn263 compared to the wt strain. These data are consistent with previous observations that show the induction of expression of some genes in the *E. coli* biofilm, such as *dnaK* [18]. The *ptsI* gene expression is reduced in the wt strain. As PtsI serves as a gateway for phosphoenolpyruvate (PEP), it is likely that the transcription of PtsI might be reduced once PEP is transported through PtsI. In contrast, the slower metabolism of the mutant strain may still contain PEP that could be transported by PtsI, whose mRNA remains active. The *adhE* gene had lower expression in the Tn263 mutant than in the wt strain. The transcription of this gene is induced under anaerobic conditions and regulated at the transcriptional and translational levels by NADH/NAD^+^ and RNase III, respectively. For this reason, the mRNA transcripts remain at low rates in Tn263 by this regulation at transcriptional and translational levels [65,68] 

Finally, to detect whether these transcriptional changes were related to a biofilm-deficient phenotype, the genes with statistical significance were tested on biofilm-forming and non-biofilm-forming clinical strains. The results show disparity with those found in wt and Tn263. All genes reported equal expression in either group of clinical isolates. Among these nine genes, only two were significant (*adhE* and *pta*) and *pta* was the only one that followed the same pattern for both Tn263 and the non-biofilm-forming strains. This may be due to the insertion of the transposon in *purL* rather than being associated with a specific biofilm-forming or non-biofilm forming phenotype. 

In conclusion, a mutation in the *purL* gene causes defective biofilm formation, which is related to the inability to form curli fibers, thus suggesting that this gene is essential for biofilm formation in *E. coli*. The proteomic study revealed pathways that belong to expressed biofilm factors, although the *E. coli* mutant was not able to form a biofilm. Given this reduction in biofilm formation and the influence of the biofilm on chronic infections, the identification of the *purL* gene as a target gene would contribute to understanding of the mechanisms of biofilm production and to develop new antibiofilm treatments against biofilm-related infections caused by this microorganism.

## Figures and Tables

**Table 1:** Phenotypical results of selected mutants.

Sample	Swimming	Curli Production	Hemagglutination (Log_2_)
Tn5	+	+	10
Tn29	+	+	10
Tn42	+	+	10
Tn82	+	+	10
Tn90	+	+	10
Tn119	+	+	10
Tn143	+	+	10
Tn 249	+	+	10
Tn 251	+	+	10
Tn 262	+	+	10
Tn263	+	-	10
Tn 337	+	+	10
Tn 373	+	+	10
Tn 406	+	+	10
Tn 420	+	+	10
Tn 425	+	+	10
Tn 457	+	+	10
Tn 463	+	-	10
Tn 467	+	+	10
Tn 474	+	+	10

+: the mutant strain presents this characteristic; -: the mutant strain does not present this characteristic.

**Table 2:** Colony-forming units and Live/Dead BacLight Bacterial Viability assay.

	Log_10_ CFU/mL ± SD		Live/Dead		
	Biofilm	Planktonic	Integrated Density	Live (% ± SD)	Dead (% ± SD)
wild type	9.09 ± 0.24	8.99 ± 0.05	2.03 × 10^7^ ± 4.86 × 10^6^	85.40 ± 6.30	14.60 ± 6.30
Tn263	6.40 ± 0.51	6.96 ± 0.01	9.69 × 10^6^ ± 2.79 × 10^6^	86.32 ± 4.01	13.68 ± 4.01
*ΔpurL::cat*	6.22 ± 0.44	7.02 ± 0.02	1.11 × 10^7^ ± 2.34 × 10^6^	80.38 ± 6.88	19.62 ± 6.88

## Supplementary Materials

The following are available online at https://www.mdpi.com/2076-0817/9/9/774/s1, Table S1: Biofilm classification of mutants. Low biofilm former (LF), high biofilm former (HF), biofilm former (F), Table S2: Primers used in the disruption and complementation of the *purL* gene, Table S3: Antibiotic resistance profiles of biofilm-forming and non-biofilm-forming clinical isolates, Table S4: qPCR primers used in this study, Table S5: Protein identification analysed by liquid chromatography coupled to mass spectrometry, Figure S1: Swimming assay. Figure S2: Two dimensional SDS-PAGE images of the wild type strain, Figure S3: Two dimensional SDS-PAGE images of the Tn263 mutant.
