# Supplementary material for: Transposon Insertion in the purL Gene Induces Biofilm Depletion in Escherichia coli ATCC 25922
Source: Pathogens. 2020 Sep 22;9(9):774. doi: 10.3390/pathogens9090774 (PMC7558270; doi:10.3390/pathogens9090774)
Supplement: Supplementary file 1 [file pathogens-09-00774-s001.pdf]

**Table S1.** Biofilm classification of mutants. Low biofilm former (LF), high biofilm former (HF), biofilm former (F).

| Samples | Significant | Adjusted *p* value | Mean $OD_{580nm}$ | Classification |
|---|---|---|---|---|
| Tn 1 | No | >0.9999 | 0.9418 | F |
| Tn 2 | No | 0.0505 | 1.414 | F |
| Tn 3 | No | 0.9976 | 0.7536 | F |
| Tn 4 | No | >0.9999 | 1.017 | F |
| Tn 5 | Yes | 0.0090 | 0.541 | LF |
| Tn 6 | No | 0.9645 | 0.7268 | F |
| Tn 7 | No | 0.0786 | 0.5996 | F |
| Tn 8 | No | >0.9999 | 0.8746 | F |
| Tn 9 | No | >0.9999 | 0.7888 | F |
| Tn 10 | No | 0.2761 | 0.642 | F |
| Tn 11 | No | >0.9999 | 1.039 | F |
| Tn 12 | No | 0.1612 | 1.377 | F |
| Tn 13 | No | >0.9999 | 0.9573 | F |
| Tn 14 | No | >0.9999 | 1.1 | F |
| Tn 15 | No | >0.9999 | 1.08 | F |
| Tn 16 | No | >0.9999 | 1.051 | F |
| Tn 17 | No | 0.6734 | 0.6844 | F |
| Tn 18 | No | >0.9999 | 0.8756 | F |
| Tn 19 | No | >0.9999 | 0.8105 | F |
| Tn 20 | No | >0.9999 | 0.8673 | F |
| Tn 21 | No | >0.9999 | 0.9304 | F |
| Tn 22 | No | >0.9999 | 1.04 | F |
| Tn 23 | No | >0.9999 | 0.8415 | F |
| Tn 24 | No | >0.9999 | 0.8843 | F |
| Tn 25 | No | >0.9999 | 0.8656 | F |
| Tn 26 | No | 0.9996 | 0.7655 | F |
| Tn 27 | No | >0.9999 | 0.8644 | F |
| Tn 28 | Yes | <0.0001 | 1.631 | HF |
| Tn 29 | Yes | <0.0001 | 0.3125 | LF |
| Tn 30 | No | >0.9999 | 0.9574 | F |
| Tn 31 | No | >0.9999 | 1.202 | F |
| Tn 32 | No | 0.5701 | 1.326 | F |
| Tn 33 | No | >0.9999 | 1.096 | F |
| Tn 34 | No | 0.5113 | 1.332 | F |
| Tn 35 | No | 0.9229 | 0.716 | F |
| Tn 36 | No | >0.9999 | 0.7819 | F |
| Tn 37 | No | 0.3684 | 1.347 | F |
| Tn 38 | No | >0.9999 | 1.176 | F |
| Tn 39 | No | >0.9999 | 1.196 | F |
| Tn 40 | No | >0.9999 | 1.07 | F |
| Tn 41 | No | 0.1929 | 0.6289 | F |
| Tn 42 | Yes | <0.0001 | 0.3976 | LF |
| Tn 43 | No | >0.9999 | 1.125 | F |
| Tn 44 | No | 0.1929 | 0.6289 | F |
| Tn 45 | No | 0.9997 | 1.232 | F |
| Tn 46 | No | >0.9999 | 0.9839 | F |

| | | | | |
|---|---|---|---|---|
| Tn 47 | No | 0.7561 | 1.307 | F |
| Tn 48 | No | >0.9999 | 0.8361 | F |
| Tn 49 | No | >0.9999 | 0.9037 | F |
| Tn 50 | Yes | <0.0001 | 1.569 | HF |
| Tn 51 | No | >0.9999 | 1.111 | F |
| Tn 52 | No | >0.9999 | 0.8481 | F |
| Tn 53 | No | 0.2689 | 1.359 | F |
| Tn 54 | No | >0.9999 | 0.9864 | F |
| Tn 55 | No | >0.9999 | 0.9508 | F |
| Tn 56 | No | >0.9999 | 0.9251 | F |
| Tn 57 | No | 0.9981 | 0.7555 | F |
| Tn 58 | No | >0.9999 | 1.132 | F |
| Tn 59 | No | 0.8034 | 1.302 | F |
| Tn 60 | Yes | 0.0240 | 1.434 | HF |
| Tn 61 | No | >0.9999 | 1.137 | F |
| Tn 62 | No | 0.9998 | 1.23 | F |
| Tn 63 | No | >0.9999 | 0.8068 | F |
| Tn 64 | No | >0.9999 | 1.196 | F |
| Tn 65 | No | >0.9999 | 1.17 | F |
| Tn 66 | No | >0.9999 | 1.185 | F |
| Tn 67 | No | 0,5319 | 1.33 | F |
| Tn 68 | No | 0,7306 | 1.31 | F |
| Tn 69 | No | >0,9999 | 1.036 | F |
| Tn 70 | No | 0,9999 | 0.7726 | F |
| Tn 71 | Yes | <0,0001 | 1.565 | HF |
| Tn 72 | No | 0,3906 | 1.344 | F |
| Tn 73 | Yes | 0,0065 | 1.467 | HF |
| Tn 74 | No | >0,9999 | 0.9809 | F |
| Tn 75 | No | >0,9999 | 0.9871 | F |
| Tn 76 | No | >0.9999 | 1.217 | F |
| Tn 77 | No | >0.9999 | 1.127 | F |
| Tn 78 | No | 0.9851 | 1.263 | F |
| Tn 79 | No | >0.9999 | 1.091 | F |
| Tn 80 | No | 0.8552 | 0.7051 | F |
| Tn 81 | No | >0.9999 | 1.129 | F |
| Tn 82 | Yes | <0.0001 | 0.3263 | LF |
| Tn 83 | No | >0.9999 | 0.8105 | F |
| Tn 84 | No | 0.1587 | 1.378 | F |
| Tn 85 | No | >0.9999 | 0.923 | F |
| Tn 86 | No | 0.9619 | 0.7258 | F |
| Tn 87 | No | >0.9999 | 0.7904 | F |
| Tn 88 | No | >0.9999 | 1 | F |
| Tn 89 | No | >0.9999 | 0.9833 | F |
| Tn 90 | Yes | 0.0423 | 0.5813 | LF |
| Tn 91 | No | 0.6697 | 0.6837 | F |
| Tn 92 | No | 0.3059 | 1.354 | F |
| Tn 93 | No | >0.9999 | 0.9579 | F |
| Tn 94 | No | >0.9999 | 0.8804 | F |
| Tn 95 | No | 0.2311 | 0.6354 | F |
| Tn 96 | No | >0.9999 | 1.012 | F |

| | | | | |
|---|---|---|---|---|
| Tn 97 | No | 0.7740 | 0.6947 | F |
| Tn 98 | No | >0.9999 | 0.81 | F |
| Tn 99 | No | >0.9999 | 1.152 | F |
| Tn 100 | Yes | 0.0298 | 1.428 | HF |
| Tn 101 | Yes | 0.0002 | 1.547 | HF |
| Tn 102 | No | >0.9999 | 1.141 | F |
| Tn 103 | No | 0.6478 | 1.319 | F |
| Tn 104 | No | >0.9999 | 0.8148 | F |
| Tn 105 | No | >0.9999 | 0.9852 | F |
| Tn 106 | No | >0.9999 | 0.9124 | F |
| Tn 107 | No | 0.9952 | 0.7475 | F |
| Tn 108 | No | >0.9999 | 1.202 | F |
| Tn 109 | No | >0.9999 | 1.105 | F |
| Tn 110 | No | 0.0566 | 0.5898 | F |
| Tn 111 | No | 0.1974 | 0.6298 | F |
| Tn 112 | No | 0.9997 | 0.768 | F |
| Tn 113 | No | >0.9999 | 0.8873 | F |
| Tn 114 | No | >0.9999 | 0.86 | F |
| Tn 115 | No | >0.9999 | 0.9366 | F |
| Tn 116 | No | >0.9999 | 0.9023 | F |
| Tn 117 | No | 0.9936 | 0.7452 | F |
| Tn 118 | No | >0.9999 | 1.119 | F |
| Tn 119 | Yes | <0.0001 | 0.2077 | LF |
| Tn 120 | No | >0.9999 | 0.8173 | F |
| Tn 121 | No | >0.9999 | 0.9111 | F |
| Tn 122 | No | 0.9744 | 0.7308 | F |
| Tn 123 | No | >0.9999 | 0.8578 | F |
| Tn 124 | No | >0.9999 | 1.057 | F |
| Tn 125 | No | >0.9999 | 0.7874 | F |
| Tn 126 | No | 0.1373 | 0.6171 | F |
| Tn 127 | No | 0.5092 | 0.6678 | F |
| Tn 128 | No | >0.9999 | 1.134 | F |
| Tn 129 | No | >0.9999 | 1.084 | F |
| Tn 130 | No | >0.9999 | 1.028 | F |
| Tn 131 | No | >0.9999 | 1.131 | F |
| Tn 132 | No | >0.9999 | 0.7769 | F |
| Tn 133 | No | >0.9999 | 0.8693 | F |
| Tn 134 | No | 0.9996 | 0.7662 | F |
| Tn 135 | No | >0.9999 | 0.8337 | F |
| Tn 136 | No | 0.7949 | 0.6973 | F |
| Tn 137 | No | 0.7762 | 1.305 | F |
| Tn 138 | No | 0.3267 | 0.6486 | F |
| Tn 139 | No | 0.7211 | 0.689 | F |
| Tn 140 | No | >0.9999 | 0.8135 | F |
| Tn 141 | No | 0.9825 | 0.7352 | F |
| Tn 142 | No | 0.8765 | 0.7083 | F |
| Tn 143 | Yes | 0.0474 | 0.5845 | LF |
| Tn 144 | No | >0.9999 | 0.8326 | F |
| Tn 145 | No | 0.9995 | 0.7644 | F |
| Tn 146 | No | >0.9999 | 0.966 | F |

| | | | | |
|---|---|---|---|---|
| Tn 147 | No | 0.9975 | 1.247 | F |
| Tn 148 | No | 0.8555 | 1.295 | F |
| Tn 149 | No | 0.2319 | 0.6356 | F |
| Tn 150 | No | >0.9999 | 0.8208 | F |
| Tn 151 | No | 0.1165 | 0.6117 | F |
| Tn 152 | No | >0.9999 | 0.7977 | F |
| Tn 153 | No | 0.8391 | 0.7028 | F |
| Tn 154 | No | >0.9999 | 0.8853 | F |
| Tn 155 | No | >0.9999 | 0.7961 | F |
| Tn 156 | No | >0.9999 | 0.8212 | F |
| Tn 157 | No | >0.9999 | 0.878 | F |
| Tn 158 | No | >0.9999 | 0.9997 | F |
| Tn 159 | No | >0.9999 | 0.9055 | F |
| Tn 160 | No | >0.9999 | 1.092 | F |
| Tn 161 | No | >0.9999 | 0.9177 | F |
| Tn 162 | No | 0.0632 | 0.593 | F |
| Tn 163 | No | >0.9999 | 0.8342 | F |
| Tn 164 | No | >0.9999 | 0.8217 | F |
| Tn 165 | No | >0.9999 | 0.7812 | F |
| Tn 166 | No | >0.9999 | 0.8308 | F |
| Tn 167 | No | >0.9999 | 0.8097 | F |
| Tn 168 | No | 0.2906 | 0.6439 | F |
| Tn 169 | No | >0.9999 | 0.9294 | F |
| Tn 170 | No | 0.7496 | 0.6921 | F |
| Tn 171 | No | >0.9999 | 0.9512 | F |
| Tn 172 | No | >0.9999 | 1.11 | F |
| Tn 173 | No | 0.9958 | 1.251 | F |
| Tn 174 | No | >0.9999 | 0.792 | F |
| Tn 175 | No | >0.9999 | 0.9955 | F |
| Tn 176 | No | >0.9999 | 1.083 | F |
| Tn 177 | No | >0.9999 | 0.7982 | F |
| Tn 178 | No | 0.9134 | 0.7142 | F |
| Tn 179 | No | 0.9442 | 0.7208 | F |
| Tn 180 | No | >0.9999 | 1.202 | F |
| Tn 181 | No | 0.9865 | 1.262 | F |
| Tn 182 | No | >0.9999 | 1.205 | F |
| Tn 183 | No | 0.6569 | 0.6824 | F |
| Tn 184 | Yes | <0.0001 | 1.616 | HF |
| Tn 185 | No | >0.9999 | 1.037 | F |
| Tn 186 | No | >0.9999 | 0.9482 | F |
| Tn 187 | Yes | 0.0038 | 1.48 | HF |
| Tn 188 | No | >0.9999 | 0.8744 | F |
| Tn 189 | No | >0.9999 | 1.184 | F |
| Tn 190 | No | >0.9999 | 0.8031 | F |
| Tn 191 | No | 0.9999 | 0.7737 | F |
| Tn 192 | No | >0.9999 | 0.9285 | F |

| | | | | |
|---|---|---|---|---|
| Tn 193 | No | 0.1708 | 0.6246 | F |
| Tn 194 | No | 0.0722 | 1.403 | F |
| Tn 195 | No | >0.9999 | 0.8691 | F |
| Tn 196 | No | 0.6697 | 1.316 | F |
| Tn 197 | No | 0.2281 | 1.365 | F |
| Tn 198 | Yes | 0.0001 | 1.553 | HF |
| Tn 199 | No | 0.0548 | 0.5887 | F |
| Tn 200 | Yes | <0.0001 | 1.642 | HF |
| Tn 201 | No | >0.9999 | 1.068 | F |
| Tn 202 | No | 0.1420 | 0.6183 | F |
| Tn 203 | No | >0.9999 | 1.027 | F |
| Tn 204 | No | >0.9999 | 0.9035 | F |
| Tn 205 | No | >0.9999 | 0.9966 | F |
| Tn 206 | Yes | <0.0001 | 1.601 | HF |
| Tn 207 | No | >0.9999 | 1.048 | F |
| Tn 208 | No | >0.9999 | 1.171 | F |
| Tn 209 | No | >0.9999 | 0.8618 | F |
| Tn 210 | No | 0.9998 | 1.23 | F |
| Tn 211 | No | >0.9999 | 1.1 | F |
| Tn 212 | No | >0.9999 | 1.102 | F |
| Tn 213 | No | >0.9999 | 0.9313 | F |
| Tn 214 | No | 0.9996 | 1.233 | F |
| Tn 215 | No | 0.9987 | 1.242 | F |
| Tn 216 | No | >0.9999 | 0.9684 | F |
| Tn 217 | No | >0.9999 | 0.8173 | F |
| Tn 218 | No | 0.9938 | 0.7455 | F |
| Tn 219 | No | >0.9999 | 0.8347 | F |
| Tn 220 | No | >0.9999 | 0.8508 | F |
| Tn 221 | No | >0.9999 | 0.8118 | F |
| Tn 222 | Yes | 0.0002 | 1.542 | HF |
| Tn 223 | No | 0.9996 | 0.7659 | F |
| Tn 224 | Yes | 0.0024 | 1.491 | HF |
| Tn 225 | No | 0.5434 | 0.6712 | F |
| Tn 226 | No | >0.9999 | 0.8737 | F |
| Tn 227 | Yes | <0.0001 | 1.793 | HF |
| Tn 228 | No | 0.6728 | 1.316 | F |
| Tn 229 | Yes | <0.0001 | 1.695 | HF |
| Tn 230 | No | 0.1512 | 0.6204 | F |
| Tn 231 | No | 0.1775 | 0.626 | F |
| Tn 232 | No | >0.9999 | 1.131 | F |
| Tn 233 | No | 0.9991 | 0.7604 | F |
| Tn 234 | No | 0.7334 | 0.6904 | F |
| Tn 235 | No | >0.9999 | 1.104 | F |
| Tn 236 | No | >0.9999 | 0.8326 | F |
| Tn 237 | No | >0.9999 | 0.966 | F |
| Tn 238 | No | >0.9999 | 0.8722 | F |

| | | | | |
|---|---|---|---|---|
| Tn 239 | No | >0.9999 | 0.816 | F |
| Tn 240 | No | 0.9781 | 1.267 | F |
| Tn 241 | No | 0.8489 | 0.7042 | F |
| Tn 242 | No | >0.9999 | 1.06 | F |
| Tn 243 | No | >0.9999 | 0.9854 | F |
| Tn 244 | No | >0.9999 | 0.8758 | F |
| Tn 245 | No | >0.9999 | 0.9249 | F |
| Tn 246 | No | 0.8002 | 0.698 | F |
| Tn 247 | No | 0.9833 | 0.7358 | F |
| Tn 248 | No | 0.8481 | 0.704 | F |
| Tn 249 | Yes | 0.0205 | 0.5618 | LF |
| Tn 250 | No | 0.4956 | 0.6662 | F |
| Tn 251 | Yes | 0.0203 | 0.5615 | LF |
| Tn 252 | No | >0.9999 | 0.9184 | F |
| Tn 253 | No | >0.9999 | 1.02 | F |
| Tn 254 | No | >0.9999 | 0.8566 | F |
| Tn 255 | No | 0.0548 | 0.5888 | F |
| Tn 256 | No | 0.9416 | 0.7201 | F |
| Tn 257 | No | >0.9999 | 0.81 | F |
| Tn 258 | No | 0.4995 | 0.6667 | F |
| Tn 259 | No | 0.9789 | 1.267 | F |
| Tn 260 | No | 0.9992 | 1.238 | F |
| Tn 261 | No | >0.9999 | 0.9524 | F |
| Tn 262 | Yes | 0.0125 | 0.5491 | LF |
| Tn 263 | Yes | <0.0001 | 0.1594 | LF |
| Tn 264 | No | 0.9372 | 0.7191 | F |
| Tn 265 | No | >0.9999 | 1.18 | F |
| Tn 266 | No | 0.9615 | 1.274 | F |
| Tn 267 | No | 0.5822 | 1.325 | F |
| Tn 268 | Yes | 0.0030 | 1.486 | HF |
| Tn 269 | No | 0.0526 | 1.412 | F |
| Tn 270 | No | 0.0505 | 1.414 | F |
| Tn 271 | Yes | 0.0031 | 1.485 | HF |
| Tn 272 | Yes | <0.0001 | 1.613 | HF |
| Tn 273 | No | 0.0789 | 1.4 | F |
| Tn 274 | No | 0.1165 | 1.388 | F |
| Tn 275 | Yes | 0.0322 | 1.426 | HF |
| Tn 276 | Yes | 0.0052 | 1.472 | HF |
| Tn 277 | No | 0.2132 | 1.368 | F |
| Tn 278 | No | >0.9999 | 1.055 | F |
| Tn 279 | Yes | 0.0069 | 1.466 | HF |
| Tn 280 | No | >0.9999 | 0.8721 | F |
| Tn 281 | Yes | <0.0001 | 1.613 | HF |
| Tn 282 | Yes | <0.0001 | 1.627 | HF |
| Tn 283 | Yes | 0.0012 | 1.506 | HF |
| Tn 284 | No | 0.9994 | 1.237 | F |

| | | | | |
|---|---|---|---|---|
| Tn 285 | No | 0.9959 | 1.251 | F |
| Tn 286 | Yes | <0.0001 | 1.617 | HF |
| Tn 287 | Yes | 0.0005 | 1.527 | HF |
| Tn 288 | No | 0.1764 | 1.374 | F |
| Tn 289 | Yes | <0.0001 | 1.736 | HF |
| Tn 290 | Yes | <0.0001 | 1.589 | HF |
| Tn 291 | Yes | 0.0001 | 1.553 | HF |
| Tn 292 | No | 0.1167 | 1.388 | F |
| Tn 293 | Yes | <0.0001 | 1.599 | HF |
| Tn 294 | Yes | <0.0001 | 1.594 | HF |
| Tn 295 | No | >0.9999 | 0.9814 | F |
| Tn 296 | Yes | 0.0040 | 1.479 | HF |
| Tn 297 | No | 0.0561 | 1.41 | F |
| Tn 298 | No | 0.3047 | 1.354 | F |
| Tn 299 | No | 0.9959 | 1.251 | F |
| Tn 300 | Yes | 0.0498 | 1.414 | HF |
| Tn 301 | No | 0.0742 | 1.402 | F |
| Tn 302 | Yes | 0.0020 | 1.495 | HF |
| Tn 303 | No | >0.9999 | 0.7902 | F |
| Tn 304 | Yes | <0.0001 | 1.598 | HF |
| Tn 305 | Yes | 0.0003 | 1.535 | HF |
| Tn 306 | Yes | <0.0001 | 1.692 | HF |
| Tn 307 | Yes | 0.0218 | 1.436 | HF |
| Tn 308 | No | >0.9999 | 0.9876 | F |
| Tn 309 | Yes | <0.0001 | 1.681 | HF |
| Tn 310 | Yes | <0.0001 | 1.87 | HF |
| Tn 311 | No | >0.9999 | 1.203 | F |
| Tn 312 | Yes | <0.0001 | 1.79 | HF |
| Tn 313 | Yes | <0.0001 | 1.572 | HF |
| Tn 314 | Yes | <0.0001 | 1.798 | HF |
| Tn 315 | No | 0.2281 | 1.365 | F |
| Tn 316 | No | >0.9999 | 1.16 | F |
| Tn 317 | No | 0.4982 | 1.333 | F |
| Tn 318 | No | 0.9912 | 1.258 | F |
| Tn 319 | No | 0.1330 | 0.616 | F |
| Tn 320 | No | 0.7805 | 1.304 | F |
| Tn 321 | No | 0.5445 | 0.6714 | F |
| Tn 322 | No | 0.9834 | 1.264 | F |
| Tn 323 | No | >0.9999 | 1.162 | F |
| Tn 324 | No | 0.9912 | 0.7423 | F |
| Tn 325 | No | >0.9999 | 0.9163 | F |
| Tn 326 | No | >0.9999 | 1.127 | F |
| Tn 327 | No | 0.0641 | 0.5934 | F |
| Tn 328 | No | 0.9645 | 0.7268 | F |
| Tn 329 | No | 0.6523 | 0.6818 | F |
| Tn 330 | No | >0.9999 | 1.033 | F |

| | | | | |
|---|---|---|---|---|
| Tn 331 | No | 0.9789 | 0.7333 | F |
| Tn 332 | No | >0.9999 | 0.9171 | F |
| Tn 333 | No | 0.9887 | 0.74 | F |
| Tn 334 | No | >0.9999 | 0.9863 | F |
| Tn 335 | No | >0.9999 | 1.103 | F |
| Tn 336 | No | >0.9999 | 1.17 | F |
| Tn 337 | Yes | 0.0340 | 0.5754 | LF |
| Tn 338 | No | 0.9744 | 1.269 | F |
| Tn 339 | No | 0.9997 | 0.7672 | F |
| Tn 340 | No | >0.9999 | 0.8725 | F |
| Tn 341 | No | >0.9999 | 1.101 | F |
| Tn 342 | No | >0.9999 | 1.18 | F |
| Tn 343 | No | >0.9999 | 1.134 | F |
| Tn 344 | No | >0.9999 | 1.083 | F |
| Tn 345 | No | >0.9999 | 0.7912 | F |
| Tn 346 | No | 0.1845 | 1.373 | F |
| Tn 347 | No | 0.0849 | 1.398 | F |
| Tn 348 | No | 0.1929 | 1.371 | F |
| Tn 349 | No | >0.9999 | 0.9455 | F |
| Tn 350 | Yes | 0.0070 | 1.465 | HF |
| Tn 351 | No | 0.9976 | 1.247 | F |
| Tn 352 | No | >0.9999 | 1.071 | F |
| Tn 353 | No | >0.9999 | 1.014 | F |
| Tn 354 | Yes | <0.0001 | 1.648 | HF |
| Tn 355 | Yes | <0.0001 | 1.567 | HF |
| Tn 356 | No | 0.3242 | 1.352 | F |
| Tn 357 | No | 0.4629 | 1.337 | F |
| Tn 358 | No | >0.9999 | 0.8191 | F |
| Tn 359 | No | >0.9999 | 1.099 | F |
| Tn 360 | No | >0.9999 | 0.9943 | F |
| Tn 361 | No | >0.9999 | 1.037 | F |
| Tn 362 | No | >0.9999 | 0.8578 | F |
| Tn 363 | No | 0.2380 | 1.363 | F |
| Tn 364 | No | >0.9999 | 0.9496 | F |
| Tn 365 | No | >0.9999 | 1.202 | F |
| Tn 366 | No | 0.9967 | 0.751 | F |
| Tn 367 | No | >0.9999 | 1.005 | F |
| Tn 368 | No | 0.9865 | 1.262 | F |
| Tn 369 | No | >0.9999 | 1.12 | F |
| Tn 370 | No | >0.9999 | 1.044 | F |
| Tn 371 | No | >0.9999 | 0.8264 | F |
| Tn 372 | No | 0.0980 | 0.6064 | F |
| Tn 373 | Yes | 0.0368 | 0.5775 | LF |
| Tn 374 | No | >0.9999 | 1.066 | F |
| Tn 375 | No | >0.9999 | 0.8885 | F |
| Tn 376 | No | >0.9999 | 1.058 | F |

| | | | | |
|---|---|---|---|---|
| Tn 377 | No | >0.9999 | 0.9598 | F |
| Tn 378 | No | 0.0931 | 1.395 | F |
| Tn 379 | No | >0.9999 | 1.066 | F |
| Tn 380 | No | 0.0681 | 0.5953 | F |
| Tn 381 | No | >0.9999 | 1.11 | F |
| Tn 382 | No | >0.9999 | 0.803 | F |
| Tn 383 | No | 0.9999 | 1.226 | F |
| Tn 384 | No | >0.9999 | 0.9378 | F |
| Tn 385 | No | >0.9999 | 1.136 | F |
| Tn 386 | No | >0.9999 | 0.9747 | F |
| Tn 387 | No | >0.9999 | 1.02 | F |
| Tn 388 | No | >0.9999 | 1.059 | F |
| Tn 389 | No | >0.9999 | 0.7881 | F |
| Tn 390 | No | >0.9999 | 0.8717 | F |
| Tn 391 | No | >0.9999 | 1.182 | F |
| Tn 392 | No | >0.9999 | 0.8646 | F |
| Tn 393 | No | >0.9999 | 0.8303 | F |
| Tn 394 | No | >0.9999 | 0.8419 | F |
| Tn 395 | No | >0.9999 | 0.8964 | F |
| Tn 396 | No | 0.3197 | 0.6476 | F |
| Tn 397 | No | >0.9999 | 0.958 | F |
| Tn 398 | No | >0.9999 | 0.8366 | F |
| Tn 399 | No | >0.9999 | 0.9449 | F |
| Tn 400 | No | 0.9999 | 0.7736 | F |
| Tn 401 | No | 0.1665 | 1.376 | F |
| Tn 402 | No | >0.9999 | 0.8334 | F |
| Tn 403 | No | >0.9999 | 0.8784 | F |
| Tn 404 | No | 0.8379 | 0.7025 | F |
| Tn 405 | No | 0.1446 | 0.619 | F |
| Tn 406 | Yes | 0.0125 | 0.5491 | LF |
| Tn 407 | Yes | <0.0001 | 2.08 | HF |
| Tn 408 | Yes | <0.0001 | 2.186 | HF |
| Tn 409 | No | 0.8694 | 1.293 | F |
| Tn 410 | Yes | <0.0001 | 2.424 | HF |
| Tn 411 | Yes | <0.0001 | 1.624 | HF |
| Tn 412 | No | 0.1416 | 1.382 | F |
| Tn 413 | No | >0.9999 | 1.178 | F |
| Tn 414 | No | 0.9726 | 1.27 | F |
| Tn 415 | Yes | <0.0001 | 1.891 | HF |
| Tn 416 | Yes | 0.0007 | 1.518 | HF |
| Tn 417 | Yes | <0.0001 | 1.92 | HF |
| Tn 418 | Yes | <0.0001 | 1.736 | HF |
| Tn 419 | Yes | <0.0001 | 1.58 | HF |
| Tn 420 | Yes | 0.0019 | 0.5034 | LF |
| Tn 421 | No | >0.9999 | 1.197 | F |
| Tn 422 | No | >0.9999 | 0.9082 | F |

| | | | | |
|---|---|---|---|---|
| Tn 423 | Yes | <0.0001 | 1.745 | HF |
| Tn 424 | Yes | <0.0001 | 2.217 | HF |
| Tn 425 | Yes | <0.0001 | 0.4316 | LF |
| Tn 426 | Yes | 0.0006 | 1.523 | HF |
| Tn 427 | Yes | <0.0001 | 2.354 | HF |
| Tn 428 | No | 0.6299 | 1.32 | F |
| Tn 429 | Yes | <0.0001 | 1.789 | HF |
| Tn 430 | No | >0.9999 | 0.8555 | F |
| Tn 431 | No | >0.9999 | 1.119 | F |
| Tn 432 | No | >0.9999 | 0.9895 | F |
| Tn 433 | No | 0.9369 | 1.281 | F |
| Tn 434 | No | 0.0674 | 0.5949 | F |
| Tn 435 | No | 0.0630 | 0.5928 | F |
| Tn 436 | No | 0.7146 | 0.6883 | F |
| Tn 437 | No | >0.9999 | 0.9958 | F |
| Tn 438 | No | 0.5863 | 0.6756 | F |
| Tn 439 | No | >0.9999 | 1.138 | F |
| Tn 440 | Yes | 0.0088 | 1.459 | HF |
| Tn 441 | No | >0.9999 | 0.8075 | F |
| Tn 442 | Yes | <0.0001 | 1.634 | HF |
| Tn 443 | No | >0.9999 | 0.9568 | F |
| Tn 444 | No | 0.6630 | 0.683 | F |
| Tn 445 | Yes | 0.0158 | 1.445 | HF |
| Tn 446 | No | >0.9999 | 1.124 | F |
| Tn 447 | No | 0.1152 | 1.389 | F |
| Tn 448 | No | 0.3791 | 1.345 | F |
| Tn 449 | No | >0.9999 | 1.015 | F |
| Tn 450 | No | >0.9999 | 0.9515 | F |
| Tn 451 | No | >0.9999 | 0.9357 | F |
| Tn 452 | No | 0.0735 | 0.5976 | F |
| Tn 453 | No | 0.9655 | 0.7273 | F |
| Tn 454 | No | 0.7818 | 1.304 | F |
| Tn 455 | No | 0.0892 | 0.6034 | F |
| Tn 456 | No | >0.9999 | 0.8051 | F |
| Tn 457 | Yes | 0.0393 | 0.5793 | LF |
| Tn 458 | No | >0.9999 | 0.8425 | F |
| Tn 459 | Yes | 0.0038 | 1.48 | HF |
| Tn 460 | No | >0.9999 | 1.061 | F |
| Tn 461 | No | >0.9999 | 1.029 | F |
| Tn 462 | No | 0.2373 | 0.6365 | F |
| Tn 463 | Yes | <0.0001 | 0.06496 | LF |
| Tn 464 | No | >0.9999 | 1.078 | F |
| Tn 465 | Yes | <0.0001 | 1.6 | HF |
| Tn 466 | No | >0.9999 | 0.9096 | F |
| Tn 467 | Yes | 0.0028 | 0.5127 | LF |
| Tn 468 | Yes | <0.0001 | 1.566 | HF |

| | | | | |
|---|---|---|---|---|
| Tn 469 | No | >0.9999 | 1 | F |
| Tn 470 | No | >0.9999 | 0.916 | F |
| Tn 471 | No | >0.9999 | 0.951 | F |
| Tn 472 | Yes | <0.0001 | 1.672 | HF |
| Tn 473 | Yes | <0.0001 | 2.222 | HF |
| Tn 474 | No | 0.0915 | 1.396 | F |
| Tn 475 | Yes | 0.0012 | 0.4925 | LF |
| Tn 476 | Yes | <0.0001 | 2.135 | HF |
| Tn 477 | No | >0.9999 | 0.9569 | F |
| Tn 478 | No | >0.9999 | 1.179 | F |
| Tn 479 | Yes | 0.0286 | 1.429 | HF |
| Tn 480 | No | 0.0893 | 1.396 | F |
| Tn 481 | No | >0.9999 | 0.9785 | F |
| Tn 482 | No | >0.9999 | 1.061 | F |
| Tn 483 | No | 0.9957 | 1.251 | F |
| Tn 484 | No | >0.9999 | 0.9806 | F |
| Tn 485 | No | 0.8873 | 1.29 | F |
| Tn 486 | No | >0.9999 | 1.193 | F |
| Tn 487 | No | 0.9990 | 1.24 | F |
| Tn 488 | Yes | <0.0001 | 2.271 | HF |
| Tn 489 | Yes | 0.0033 | 1.483 | HF |
| Tn 490 | Yes | <0.0001 | 2.232 | HF |
| Tn 491 | No | >0.9999 | 1.033 | F |
| Tn 492 | Yes | <0.0001 | 2.767 | HF |
| Tn 493 | Yes | 0.0006 | 1.523 | HF |
| Tn 494 | Yes | <0.0001 | 1.83 | HF |
| Tn 495 | Yes | <0.0001 | 1.68 | HF |
| Tn 496 | Yes | <0.0001 | 1.816 | HF |
| Tn 497 | Yes | <0.0001 | 2.567 | HF |
| Tn 498 | Yes | 0.0001 | 1.555 | HF |
| Tn 499 | Yes | <0.0001 | 2.551 | HF |
| Tn 500 | Yes | <0.0001 | 1.721 | HF |
| Tn 501 | No | 0.1248 | 1.386 | F |
| Tn 502 | No | >0.9999 | 0.9037 | F |
| Tn 503 | No | >0.9999 | 0.8236 | F |
| Tn 504 | No | >0.9999 | 1.071 | F |
| Tn 505 | No | >0.9999 | 1.112 | F |
| Tn 506 | No | >0.9999 | 0.8893 | F |
| Tn 507 | Yes | 0.0002 | 1.549 | HF |
| Tn 508 | No | 0.1373 | 1.383 | F |
| Tn 509 | Yes | <0.0001 | 1.725 | HF |
| Tn 510 | No | >0.9999 | 0.9944 | F |
| Tn 511 | No | >0.9999 | 0.867 | F |
| Tn 512 | No | >0.9999 | 1.189 | F |
| Tn 513 | Yes | 0.0003 | 1.536 | HF |
| Tn 514 | No | >0.9999 | 1.1 | F |

| | | | | |
|---|---|---|---|---|
| Tn 515 | Yes | <0.0001 | 1.822 | HF |
| Tn 516 | No | 0.9982 | 1.244 | F |
| Tn 517 | No | 0.1309 | 1.385 | F |
| Tn 518 | No | >0.9999 | 0.8848 | F |
| Tn 519 | Yes | <0.0001 | 1.577 | HF |
| Tn 520 | Yes | <0.0001 | 1.874 | HF |
| Tn 521 | No | 0.5640 | 1.327 | F |
| Tn 522 | Yes | 0.0036 | 1.481 | HF |
| Tn 523 | Yes | <0.0001 | 2.009 | HF |
| Tn 524 | No | 0.1698 | 1.376 | F |
| Tn 525 | Yes | <0.0001 | 1.918 | HF |
| Tn 526 | Yes | <0.0001 | 1.717 | HF |
| Tn 527 | Yes | 0.0001 | 1.556 | HF |
| Tn 528 | No | 0.5504 | 0.672 | F |
| Tn 529 | No | 0.9561 | 1.276 | F |
| Tn 530 | No | >0.9999 | 1.015 | F |
| Tn 531 | No | >0.9999 | 0.9497 | F |
| Tn 532 | No | 0.5063 | 1.333 | F |
| Tn 533 | No | >0.9999 | 1.167 | F |
| Tn 534 | Yes | 0.0003 | 1.535 | HF |
| Tn 535 | No | 0.8578 | 1.294 | F |
| Tn 536 | Yes | <0.0001 | 2.551 | HF |

**Table S2.** Primers used in the disruption and complementation of the *purL* gene.

| Target | Primer Sequence (5' to 3') | Tm (°C) | Product size | Reference |
|---|---|---|---|---|
| cat (Chloramphenicol cassette) left border | GGGCGAAGAAGTTGTCCATA | 60 | 914 pb | This study |
| *purL* right border | GCAAACCACCCATTGTCG | 54.7 | | This study |
| *purL* left border | CGTCAGCGCATCAGATTC | 53.8 | | This study |
| cat (Chloramphenicol cassette) right border | TGGAGTGAATACCACGACGA | 60 | 511 pb | This study |
| *purL* - F | ATGATGGAAATTCTGCGTGGTTCGCCTGC | 64.6 | | This study |
| | | | 3888 pb | |
| *purL* - R | TTACCCCAACTGCTTACGCGCATTGCGGAA | 66.8 | | This study |
| T7 promoter - F | TAATACGACTCACTATAGGG | 54 | | This study |
| | | | 800 pb | |
| *purL* internal - R | CGTGGTTTCGAAGGTGTTTT | 58 | | This study |

**Table S3.** Antibiotic resistance profiles of biofilm-former and non-biofilm-former clinical isolates.

| | | Antibiotic Resistance Profiles | | | | | | | | | | | | |
|---|---|---|---|---|---|---|---|---|---|---|---|---|---|---|
| **Sample** | **Origin** | **GM** | **AMC** | **PTZ** | **SXT** | **CAZ** | **CIP** | **IMI** | **COL** | **AZT** | **FOS** | **TG** | **CHL** | **Biofilm** |
| HMT119 | Urine | S | S | S | I | S | I | S | S | S | S | S | S | LF |
| HCB156 | Urine | R | S | S | R | S | S | S | S | S | S | R | S | LF |
| HCB0006 | Blood | S | S | S | S | S | S | S | S | S | S | S | S | LF |
| HMT142 | Blood | S | S | S | S | S | S | S | S | S | S | S | S | F |
| HUB647 | Urine | S | S | S | S | S | S | S | S | S | S | S | S | F |
| HUB656 | Urine | S | S | S | S | S | S | S | S | S | S | S | S | F |

GM: Gentamicin, AMC: Amoxicillin/clavulanic acid, PTZ: Piperacillin-tazobactam, SXT: Trimethoprim- sulfamethoxazole. CAZ: Ceftazidime, CIP: Ciprofloxacin, IMI: Imipenem, COL: Colistin, FOS: Fosfomycin, TG: Tygecycline, CHL: Chloramphenicol. S: Susceptible, I: Intermediate, R: Resistant. LF: low former, F: former.

**Table S4.** qPCR primers used in this study.

| Gen | Primer sequence (5' to 3') | Tm (°C) | Product Size | Reference |
|---|---|---|---|---|
| *mgtA* | F: GTGAGTCTCTGCCCGTAGAAAAA | 63 | 71 bp | This study |
| | R: GTCGCACTCCAGCGGATT | 58 | | |
| *dnaK* | F: GCGAAACTGGAAAGCCTGGTTGAA | 65 | 136 bp | [1] |
| | R: TTGGCATACGAGTCTGACCACCAA | 65 | | |
| *lptD* | F: TGGAACATCGCGCCAAAT | 54 | 62 bp | This study |
| | R: TTGCCACGACGATGCATATAA | 57 | | |
| *ptsI* | F: CGTATCGCGATGGATCGTAGA | 61 | 63 bp | This study |
| | R: CGAGGCACGCAGGATAGC | 61 | | |
| *pta* | F: ACAATGTTGATCCGGCGAAG | 58 | 101 bp | [2] |
| | R: CATATCGATCGCACGAGTCG | 60 | | |
| *ilvC* | F: TTCTCTGCTGTGCTTCGA | 59 | 82 bp | This study |
| | R: TTCCCAACCGAACTGAATCAG | 59 | | |
| *tsf* | F: TGAAGGCCGCATGAAGAAA | 55 | 80 bp | This study |
| | R: CAACAGTTTTGCTTGGTTCCATAA | 60 | | |
| *ftsz* | F: TTACTCGTCCGGGTTTGATGA | 59 | 71 bp | This study |
| | R: TGCGTAGCCCATCTCAGACA | 60 | | |
| *elbB* | F: TGAAGCGGTGTTGACGTTGT | 58 | 73 bp | This study |
| | R: CTGCTTATCCGGTGCAAAGC | 60 | | |
| *groL* | F: GGGTATCCTGGATCCAACCA | 60 | 70 bp | This study |
| | R: CATCAGGCCAGCCACAGAA | 59 | | |
| *ompA* | F: CTGGTGCTAAACTGGGCTG | 59 | 112 bp | [3] |
| | R: TTAACCTGGTAACCACCAAAAG | 58 | | |
| *adhE* | F: AAGTCCCTGTGTGCTTTCGG | 60 | 101 bp | [2] |
| | R: TGCAGAGCCTGACCATCAGA | 60 | | |
| *purL* | F: CCGTAATGGAAGGTTCTGAAGTG | 63 | 112 bp | This study |
| | R: TGGTTGTGAGTTTCGACTTTCATC | 62 | | |
| *arcA* | F: CGAAATCGGTGCAGATGACTAC | 60 | 68 bp | This study |
| | R: TGCGTGCACGAATAGTCAGTT | 62 | | |

**Table S5.** Protein identification analysed by liquid chromatography coupled to mass spectrometry and their coding gene.

| SPOT | Gene name(s) | Protein cat. | Uniprot Entry | Peptides | Anova (p) | MW [kDa] | pI | Score | Seq. Coverage [%] | Spec Counts |
|---|---|---|---|---|---|---|---|---|---|---|
| 1 | ***lptD***, *imp*, *ostA* | 3 | Q1RGE3 | 12 | 0.0442 | 89.6 | 5 | 387.4 | 17 | 14 |
| 2 | ***ptsI*** | 3 | P08839 | 9 | 0.0309 | 63.5 | 4.6 | 221.4 | 12.9 | 9 |
| 3 | ***cysJ*** | 1 | Q1R7T4 | 3 | 0.0239 | 66.3 | 4.8 | 66 | 4.8 | 3 |
| 4 | ***pta*** | 1 | P0A9M8 | 6 | 0.0033 | 77.1 | 5.2 | 199.6 | 9 | 9 |
| 5 | ***tsf*** | 2 | C4ZRR2 | 14 | 0.0025 | 30.4 | 5.1 | 558.9 | 40.6 | 29 |
| 6 | ***ftsZ***, *sfiB*, *sulB* | 2 | P0A9A6 | 6 | 0.0380 | 40.3 | 4.5 | 185.9 | 21.9 | 9 |
| 7 | ***elbB***, *elb2*, *yzzB* | 1 | P0ABU5 | 1 | 0.0221 | 23 | 4.5 | 34.9 | 3.7 | 1 |
| 8 | ***groL***, *groEL* | 2 | A7ZV12 | 1 | 0.0093 | 57.3 | 4.7 | 36.6 | 1.8 | 1 |
| 9 | ***ompA***, *con*, *tolG*, *tut* | 3 | P0A910 | 1 | 0.0250 | 37.2 | 6 | 26.7 | 2.6 | 1 |
| 10 | ***adhE***, *ana* | 1 | P0A9Q7 | 1 | 0.0025 | 96.1 | 6.3 | 47.2 | 1.1 | 1 |
| 11 mixture | ***mgtA***, *corB*, *mgt* | | P0ABB8 | 1 | 0.0039 | 99.4 | 5.6 | 117.2 | 1.8 | 6 |
| 11 mixture | *dnaK* | 2 | A7ZVV7 | 3 | 0.0039 | 69.1 | 4.7 | 62.3 | 4.7 | 4 |
| 12 mixture | ***dnaK*** | | A7ZVV7 | 3 | 0.0024 | 69.1 | 4.7 | 62.3 | 4.7 | 4 |
| 12 mixture | *mgtA*, *corB*, *mgt* | 1 | P0ABB8 | 1 | 0.0024 | 99.4 | 5.6 | 42.4 | 1.8 | 3 |
| 13 mixture | ***ilvC*** | 1 | C4ZZ44 | 2 | 0.0018 | 54 | 5.1 | 28.8 | 3.5 | 2 |
| 13 mixture | *ygiM* | | P0ADT8 | 1 | 0.0018 | 23.1 | 9.6 | 24.6 | 4.4 | 1 |

Protein Category: 1. Metabolism and cell maintenance, 2. Signalling and cellular process, 3. Genetic information processing.

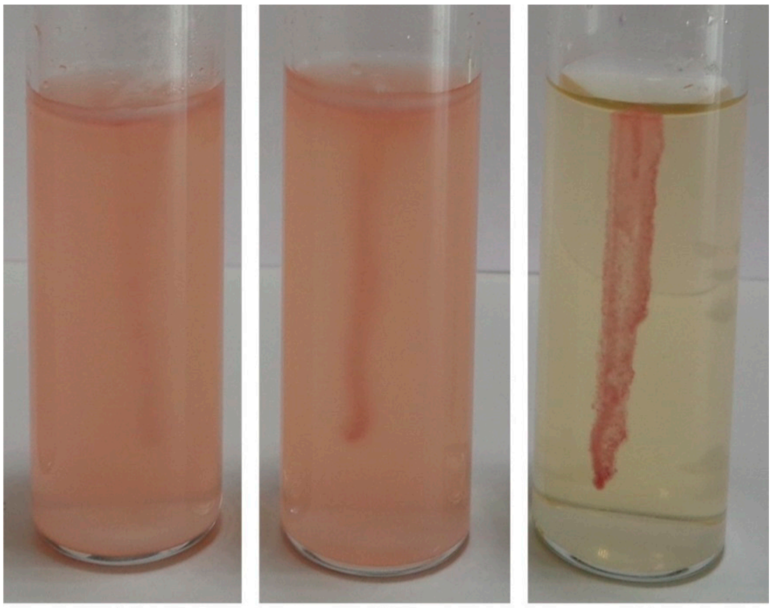

(a) (b) (c)

**Figure S1.** Swimming assay. (a): wild type; (b): Tn263; (c) *flhD* mutant as negative control.

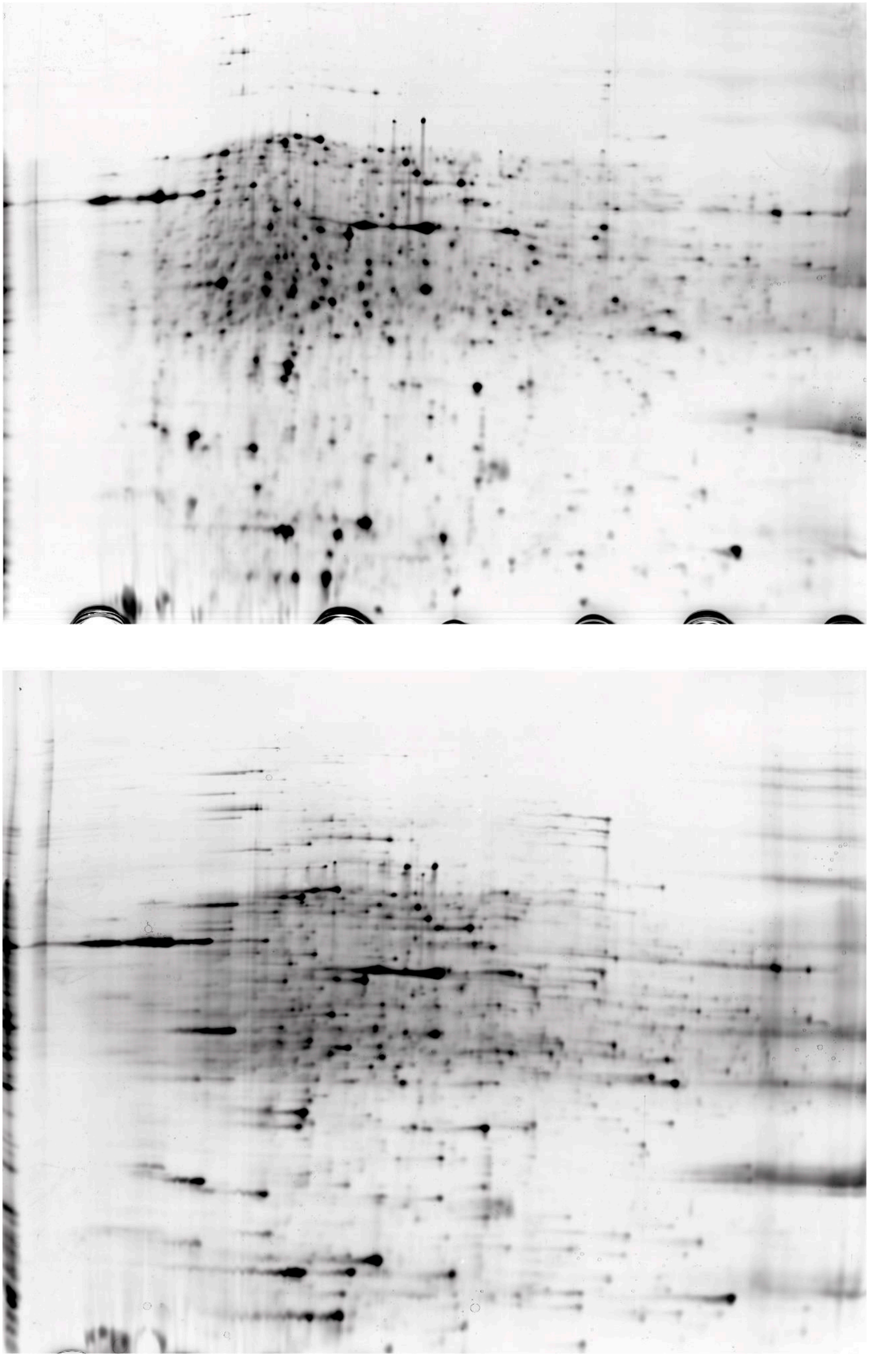

**Figure S2.** Two dimensional SDS-PAGE images of the wild type strain.

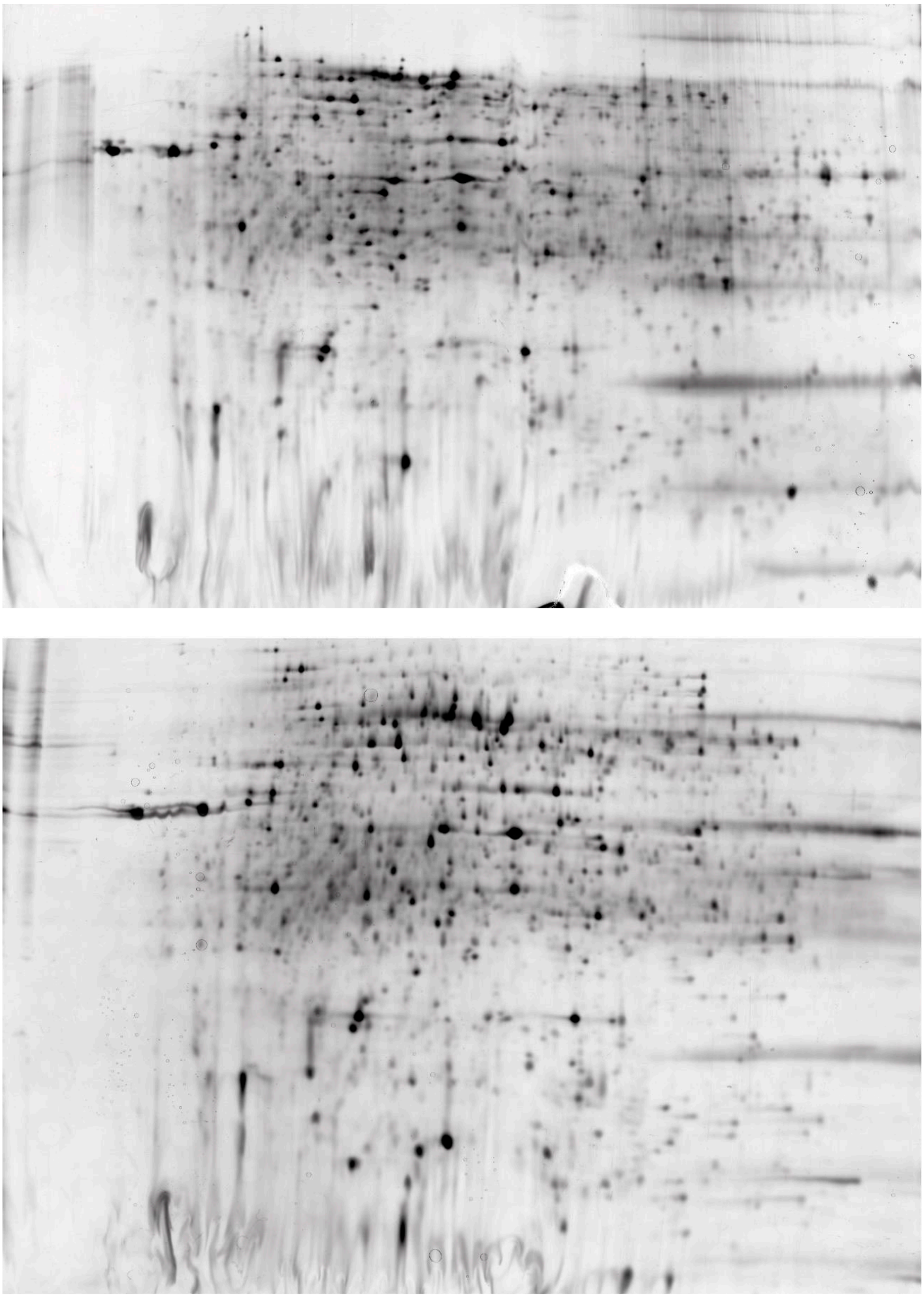

**Figure S3.** Two dimensional SDS-PAGE images of the Tn263 mutant.

## References


1. Singh, R.; Jiang, X. Expression of Stress and Virulence Genes in *Escherichia coli* O157:H7 Heat Shocked in Fresh Diary Compost. *J. Food Prot.* **2015**, *78*, 31–41.
2. Flores, N.; Flores, S.; Escalante, A.; De Anda, R.; Leal, L.; Malpica, R.; Georgellis, D.; Gosset, G.; Bolívar, F. Adaptation for fast growth on glucose by differential expression of central carbon metabolism and gal

regulon genes in an *Escherichia coli* strain lacking the phosphoenolpyruvate:carbohydrate phosphotransferase system. *Metab. Eng.* **2005**, *7*, 70–87.

3. Camprubí-Font, C.; Ruiz del Castillo, B.; Barrabés, S.; Martínez-Martínez, L.; Martinez-Medina, M. Amino Acid Substitutions and Differential Gene Expression of Outer Membrane Proteins in Adherent-Invasive *Escherichia coli*. *Front. Microbiol.* **2019**, *10*, 1–10.
